# Sensing with discrete time crystals

**DOI:** 10.1038/s41567-025-03163-6

**Published:** 2026-02-23

**Authors:** Leo Joon Il Moon, Paul M. Schindler, Ryan J. Smith, Emanuel Druga, Zhuo-Rui Zhang, Marin Bukov, Ashok Ajoy

**Affiliations:** 1 Department of Chemistry, University of California, Berkeley, Berkeley, CA USA; 2 Chemical Sciences Division, Lawrence Berkeley National Laboratory, Berkeley, CA USA; 3 Max Planck Institute for the Physics of Complex Systems, Dresden, Germany; 4 CIFAR Azrieli Global Scholars Program, Toronto, Ontario Canada

**Keywords:** Quantum metrology, Statistical physics, thermodynamics and nonlinear dynamics, Condensed-matter physics

## Abstract

Prethermal discrete time crystals are non-equilibrium states of matter with long-range spatiotemporal order and a subharmonic response stabilized by many-body interactions under periodic driving. The robustness of time-crystalline order to perturbations in the drive protocol makes these systems attractive for quantum sensing. Here we exploit the sensitivity of prethermal discrete time crystal order to deviations in its order parameter to implement the frequency-selective detection of time-varying magnetic fields in a system of strongly driven, dipolar-coupled ^13^C nuclear spins in a diamond. Incorporating an oscillating field into the time crystal dynamics extends its lifetime exponentially, producing a sharp resonant response in the order parameter. The sensor linewidth is set by the time crystal lifetime alone, as strong interspin interactions help stabilize the time-crystalline order. The device operates in the 0.5–50-kHz range—a challenging frequency regime for sensors based on atomic vapour or electronic spins—and achieves competitive sensitivity. The sensing principle we demonstrate is robust to drive errors and sample inhomogeneities, and is applicable across a range of physical platforms including superconducting circuits, neutral atoms and trapped ions.

## Abstract (web summary)

It is shown that an a.c. field exponentially extends the lifetime of a prethermal time crystal realized with nuclear spins in diamond, enabling a narrowband detection of magnetic fields.

## Main

Non-equilibrium matter has emerged as a frontier in modern many-body physics, displaying novel phenomena beyond restrictions imposed by thermal equilibrium. A milestone is the demonstration^1–13^ of discrete time crystals (DTCs)^14–22^, a new form of non-equilibrium matter that breaks time-translation symmetry, akin to ordinary crystals breaking spatial symmetry. A hallmark of DTCs is their robust period-doubling response, stabilized by many-body interactions of mean strength *J*, making them resilient to errors in the protocol creating them. Most observed time-crystalline states rely on Floquet prethermalization^23–28^, where periodically driven quantum states are preserved for durations parametrically controlled by the drive frequency, resulting in lifetimes ${T}_{2}^{{\prime} }$ far exceeding the system’s interaction-dominated intrinsic decay time ${T}_{2}^{* }$ (∝*J*^−1^).

The robustness and long lifetimes of DTCs in the presence of interactions make them promising for quantum technologies, such as simulating complex systems^29^, topologically protected quantum computation^30^ and robust generation of entangled states^31^. Experimental work has demonstrated the use of continuous time crystals as a d.c. field sensor^32^. Separately, theoretical proposals have suggested using DTCs for enhanced quantum sensing^33–37^. However, experimental realizations using DTCs as quantum sensors have been challenging due to the necessity of using strongly correlated states^33^ or fine-tuned systems^34–36^.

In this work, we develop a new approach for using DTCs to construct highly frequency-selective quantum sensors for time-varying (a.c.) magnetic fields, and demonstrate it experimentally in an ensemble of randomly positioned, hyperpolarized ^13^C nuclear spins in diamond. The sensor operates in the 0.5–50-kHz range—typically a challenging frequency regime for sensors based on atomic vapour^38^ or electronic spins^39^—and achieves competitive sensitivity. The scheme leverages the robustness of DTC order, requires no preparation of strongly correlated states and is broadly applicable in platforms exhibiting prethermal *U*(1) DTC order^2,4,5,7,10,12^.

Our approach is based on the observation that under certain conditions, the stability of DTC order can be remarkably enhanced by the presence of an a.c. field; this stabilization is highly frequency selective, enabling the DTC to be exploited for sensing. Specifically, the a.c. field *B*_a.c._(*t*) couples to the DTC order parameter only when its frequency ${f}_{{\rm{a}}.{\rm{c}}}={f}_{{\rm{r}}{\rm{e}}{\rm{s}}}$ matches the DTC oscillations, protecting the DTC order from symmetry-breaking perturbations and exponentially enhancing its lifetime ${T}_{2}^{{\prime} }$ (ref. ^40^). We demonstrate that the lifetime extension can be as much as three orders of magnitude, yielding a record for coupling-normalized DTC lifetimes $J{T}_{2}^{{\prime} }\approx 14,051$, and importantly that it is a strongly resonant effect, producing a narrow a.c. frequency response, with linewidths under 70 mHz around ${f}_{res}$, determined solely by ${({T}_{2}^{{\prime} })}^{-1}$.

We leverage this, using a DTC excited via two-tone Floquet drive^10^, to construct a noise-rejected, continuously interrogated a.c. sensor that operates for extended periods without reinitialization. Unlike traditional quantum sensing^41^, which avoids interactions between sensor spins^42^, the DTC sensor here intimately relies on these interactions, as well as thermalization, to establish a ${T}_{2}^{{\prime} }$-limited sensor linewidth, and remaining robust against drive errors and on-site disorder (Supplementary Section 7). We additionally demonstrate that the a.c.-field-mediated lifetime extension applies equally to DTCs excited along both transverse and longitudinal axes, suggesting wide applicability across diverse platforms including spin systems^2,4,5,7,10,12^, superconducting qubits^11,31,43^ and cold atoms^1,3^.

## Results

### Principle: prethermal DTC lifetime extension by a.c. fields

**Fig. 1:**
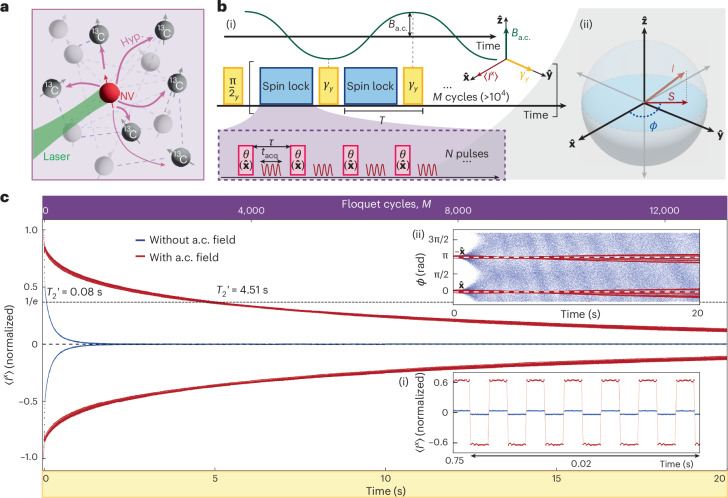
System and principle. **a**, System consists of dipolar interacting ^13^C nuclear spins, hyperpolarized by NV centres using optical and chirped microwave excitation. **b**, Protocol. (i) Evolution under native dipole–dipole interactions, ${{\mathcal{H}}}_{dd}$ (see the main text) is interrupted by a concatenated two-tone drive with *N* spin-lock (pink) $\theta (\widehat{{\bf{x}}})$ pulses separated by *τ*, interspersed with spin-flip$\gamma (\widehat{{\bf{y}}})$ pulses (yellow). This time block (total period, *T*) is repeated *M* times. The protocol causes switching between $\widehat{{\bf{x}}}\leftrightarrow -\widehat{{\bf{x}}}$ every *t* = *Nτ*, remaining robust against deviations *γ*_*y*_ = π + *ϵ*, forming a PDTC. Additionally, a $\widehat{{\bf{z}}}$-oriented a.c. field (green) with amplitude *B*_a.c._ and frequency *f*_a.c._ is applied; the resonant case ${f}_{AC}={f}_{res}=1/(2T)$ is shown. (ii) Net spin magnetization *I* is monitored during acquisition time *t*_acq_ ≈ 13.6 μs between the *θ*_*x*_ pulses. Projection *S* onto the ^13^C nuclear spin’s rotating frame $\widehat{{\bf{x}}}-\widehat{{\bf{y}}}$ plane and its phase *ϕ* are measured. **c**, Main result. Magnetization 〈*I*^*x*^〉 for the PDTC protocol without (blue) and with (red) applied resonant a.c. field. Here *N* = 16; pulse separation *τ* (36 μs); (π/2)_*y*_ and *θ*_*x*_ pulses (~50.25 μs); *γ*_*y*_ pulse (~98.5 μs); *B*_a.c._ = 82.4 μT with ${f}_{res}=330$ Hz. The top axis indicates the number of flips *M*. The dashed line indicates the 1/*e* intercept, yielding lifetimes of ${T}_{2}^{{\prime} }=80\,ms$ without an a.c. field and ${T}_{2}^{{\prime} }=4.51\,s$ with an a.c. field. Data show *M* > 13,000 〈*I*^*x*^〉 flips sustained over *t* = 20 s. (i) Zoomed-in view of the data in a small 20-ms window at *t* = 0.76 s, displaying magnetization switching from $-\widehat{{\bf{x}}}$ to $\widehat{{\bf{x}}}$. Lifetime extension under a.c. field is evident from the increased amplitude of the red data points. (ii) Tracked phase *ϕ* for data from the main panel, displaying a coherent signal far beyond the 1/*e* decay time. $\widehat{{\bf{x}}}\,(-\widehat{{\bf{x}}})$ rails correspond to phases 0 (π), respectively. Decoherence in the non-a.c. case leads to *ϕ* spread uniformly in [−π, π] (blue data points). The a.c. field leads to a notable lifetime increase (red).

The system consists of a diamond crystal with ^13^C nuclear spins hyperpolarized by optically pumped nitrogen-vacancy (NV) centres (Fig. 1a). The ^13^C nuclei, at natural abundance, are randomly distributed and influenced by fluctuating fields from lattice NV and P1 centres^44,45^. Spins interact via magnetic dipole interactions, ${{\mathcal{H}}}_{dd}={\sum }_{k < l}{J}_{kl}(3{I}_{k}^{z}{I}_{l}^{z}-{{\bf{I}}}_{k}\cdot {{\bf{I}}}_{l})$, with spin–spin coupling strengths *J*_*kl*_, and median coupling strength *J* ≈ 0.6 kHz (ref. ^46^) determined via free induction decay; ${I}_{k}^{\alpha }$ are spin-1/2 operators for ^13^C nuclear spin *k* and the total polarization is ${I}^{\alpha }={\sum }_{k}{I}_{k}^{\alpha }$, *α* = *x*, *y*, *z*.

A *U*(1) prethermal DTC (PDTC) is created using the two-tone drive protocol from ref. ^10^ (Fig. 1b(i)). Hyperpolarization initializes the ^13^C nuclear spins in the $\widehat{{\bf{x}}}$-polarized state *ρ*_0_ ∝ *I*^*x*^, after which the two-tone drive is activated. The first spin-lock drive, consisting of $\widehat{{\bf{x}}}$-oriented *θ* pulses separated by period *τ*, realizes an effective Hamiltonian *H*_eff_ with emergent *U*(1) symmetry: [*H*_eff_, *I*^*x*^] = 0.

The second drive (period *T*) establishes the PDTC order via superimposed $\widehat{{\bf{y}}}$ pulses of angle *γ*_*y*_ (~π), applied after every *N*th spin-lock pulse. The ^13^C nuclear spins are inductively interrogated between pulses via a radio-frequency cavity; a downsampling technique^47^ (Methods) enables the quasi-continuous monitoring of their projection onto the $\widehat{{\bf{x}}}\,-\,\widehat{{\bf{y}}}$ plane directly in the rotating frame (Fig. 1b(ii)). Net projection is denoted as *S*, and the phase in the $\widehat{{\bf{x}}}\,-\,\widehat{{\bf{y}}}$ plane is *ϕ* (Fig. 1b(ii)). Continuous interrogation with the two-tone drive allows full time-trace read-out in a single shot (key for quantum sensing) and distinguishes it from single-tone drives commonly used in other systems^4,8,12^ (Supplementary Section IIC).

PDTC order, arising from emergent *U*(1) symmetry, is characterized by robust period doubling, seen in the long-lived oscillation of polarization 〈*I*^*x*^〉 with period 2*T* (ref. ^10^). This is shown in Fig. 1c (blue data points), with the number of cycles *M* of the DTC *γ*_*y*_ drive on the top axes. The decay of 〈*I*^*x*^〉 has a characteristic 1/*e* time, ${T}_{2}^{{\prime} }=79\,ms$, which is much longer than ${T}_{2}^{* }=1.5\,ms$ (ref. ^46^). The purple data points in Fig. 1c(i) (inset) provide a zoomed-in view at *t* = 0.75 s.

The PDTC decay can be understood by noting that the initial state (${\rho }_{0} \sim {\mathbb{1}}+\mu {I}^{x}$) corresponds to a zero-energy state with respect to the effective Hamiltonian, ${\langle {H}_{e\mathrm{ff}}\rangle }_{{\rho }_{0}}=0$. The eigenstate thermalization hypothesis (ETH)^48–51^ implies that without conservation laws, the system prethermalizes to a featureless infinite-temperature (${\mathcal{T}}=\infty$) state, ${\rho }_{{\mathcal{T}}=\infty } \sim {\mathbb{1}}$. For *U*(1) quasi-conservation, prethermalization is restricted to states with the same polarization. However, small symmetry-breaking perturbations restore prethermalization to an infinite temperature. In particular, higher-order corrections to *H*_eff_ in the two-tone drive break *U*(1) conservation, leading to an inverse decay time (heating rate) *Γ*_e_ = 1/*T*_e_ ∝ (*JT*)^2^ (refs. ^10,46^; Supplementary Section 6).

We now consider the effect of a resonant a.c. magnetic field, *B*_a.c._(*t*)*I*^*z*^, with frequency ${f}_{res}$, aligned along $\widehat{{\bf{z}}}$ and locked to the DTC *γ*_*y*_ kicks (Fig. 1b(i), green line). We show (Supplementary Section 6) that it exponentially extends the lifetime of the *U*(1) PDTC order by the Floquet engineering of a finite energy density^40^, forming the basis for the sensor operation.

To understand this, note that the a.c. field induces an effective coupling to the PDTC order parameter (−1)^*ℓ*^*I*^*x*^, that is, *H*_eff_ → *H*_eff,ℓ,a.c._ = *H*_eff_ + (−1)^*ℓ*^*BI*^*x*^, which, like the DTC response, alternates in sign for each *T*-period *ℓ*, where the the coupling *B* ∝ *B*_a.c._ is proportional to the a.c. field strength. Considering the effective Hamiltonian every even period, *H*_eff,a.c._ = *H*_eff_ + *BI*^*x*^, the DTC-ordered initial state, ${\rho }_{0} \sim {\mathbb{1}}+\mu {I}^{x}$, acquires a finite energy density, ${\langle {H}_{{\rm{e}}\mathrm{ff},{\rm{a}}.{\rm{c}}}\rangle }_{{\rho }_{0}}={\langle {H}_{{\rm{e}}\mathrm{ff}}\rangle }_{{\rho }_{0}}+{\langle {I}^{x}\rangle }_{{\rho }_{0}}\propto \mu B$, controlled by the a.c. field (Supplementary Section 6).

Thus, the a.c. field creates a finite energy density from the PDTC order, leading the PDTC to prethermalize to a finite-temperature state, ${\rho }_{{\mathcal{T}}}\propto \exp (-{H}_{e\mathrm{ff},AC}/{\mathcal{T}})$, even with symmetry-breaking perturbations (we set Boltzmann’s constant to unity). This enhances its robustness, energetically protecting the PDTC state from prethermalization to an infinite temperature, and results in a Floquet heating rate that is now exponentially suppressed in the driving period *T*, ${\varGamma }_{{\rm{e}}}^{\,\mathrm{a.c}}\propto \exp (-1/JT)$. Note that experimentally observing this exponential extension of the lifetime is challenging due to technical limitations (Supplementary Section 6C).

Figure 1c (red data points) shows the PDTC under an a.c. field with *B*_a.c._ = 82.4 μT and ${f}_{{\rm{a}}.{\rm{c}}}={f}_{{\rm{r}}{\rm{e}}{\rm{s}}}=330.023$ Hz. The 1/*e* lifetime is extended more than 50 fold to ${T}_{2}^{{\prime} }=4.51\,s$, and corresponding to over $M=2,900\,\widehat{{\bf{x}}}\leftrightarrow -\widehat{{\bf{x}}}$ spin flips. This manifests also in the larger signal in the zoomed-in view (Fig. 1c(i)). In particular, lifetime extension is not limited to the specific case of *γ*_*y*_ = π but applies throughout the stability regime of the DTC order (Supplementary Section 3 and Supplementary Fig. 2).

Spin evolution remains observable far beyond the value naively suggested by the 1/*e* time, as shown by the phase signal *ϕ* remaining coherent for several seconds (Fig. 1c(ii)). The $\widehat{{\bf{x}}}$ and $-\widehat{{\bf{x}}}$ rails correspond to phase values *ϕ* = 0, π, with each point tracking *ϕ* after every *θ* pulse (total, >500,000). Heating of the conventional PDTC (blue data points) towards the infinite-temperature state, ${\rho }_{{\mathcal{T}}=\infty }={\mathbb{1}}$, is evident as *ϕ* disperses across the [−π, π] phase space within ~2 s. By contrast, under the a.c. field (Fig. 1c(ii), red data points), the PDTC signal remains stable for over 20 s and 544,000 pulses (spin lock plus *γ* kick). We also note that with the a.c. field applied, micromotion among the interpulse spacings within a single period of the DTC sequence causes an apparent splitting of the signal into multiple strands. As described in ref. ^52^, this is due to the distinct prethermal plateaus corresponding to each stroboscopic frame within the period of the Floquet cycle. Similar micromotion is also evident in the signal shown in Supplementary Fig. 4.

### Robust, high-resolution a.c. magnetic field sensing

**Fig. 2:**
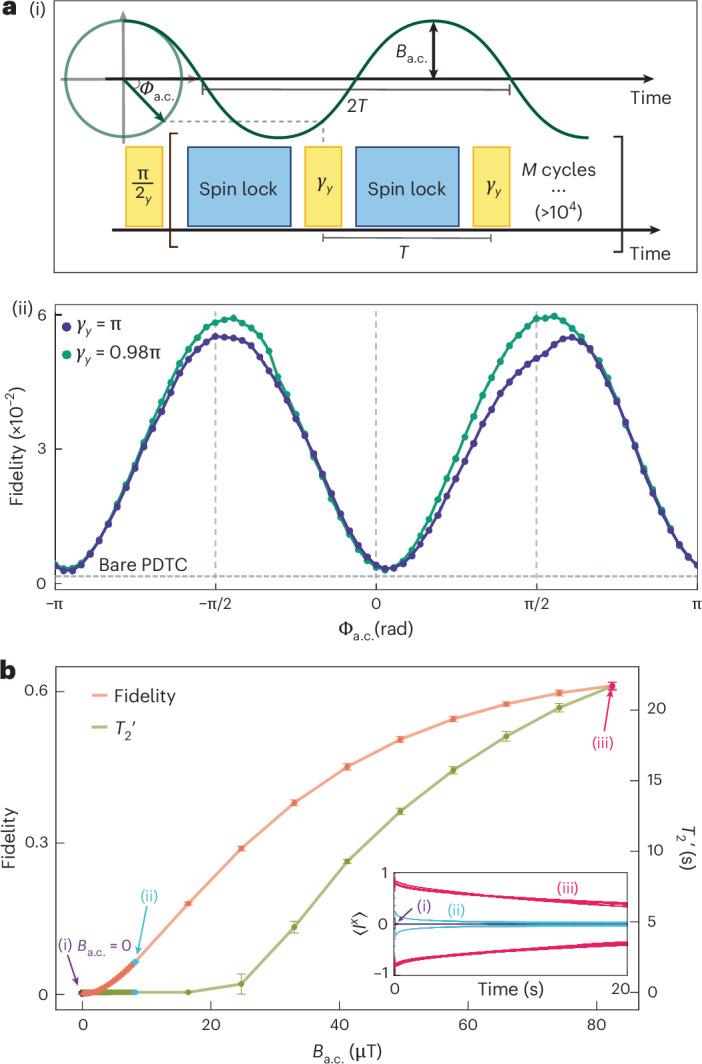
PDTC lifetime extension under resonant a.c. fields. **a**, Effect of a.c. field phase *Φ*_a.c._. (i) Schematic: a.c. field phase *Φ*_a.c._ is measured relative to the application of *γ*_*y*_ pulses, with *Φ*_a.c._ = ±π/2 indicating that the troughs and peaks of the a.c. field align with the centre of *γ*_*y*_ kicks. (ii) Lifetime extension fidelity *F* (equation (1)) as a function of the a.c. field phase *Φ*_a.c._ at a fixed amplitude of *B*_a.c._ = 8.24 μT on resonance. Signal increase is the strongest at *Φ*_a.c._ = π/2; for *Φ*_a.c._ = 0, there is minimal lifetime increase over bare PDTC (dashed line). Blue (green) data points show cases for *γ*_*y*_ pulses on (*ϵ* = 2% away from) the *γ*_*y*_ = π PDTC stable point. Qualitatively, the same behaviour is observed, indicating PDTC robustness. (π/2)_*y*_ and *θ*_*x*_ pulses (~50.25 μs), π pulse (~100.5 μs), *τ* (~36 μs) and 0.98π pulse (~98.5 μs). **b**, Effect of a.c. field amplitude *B*_a.c._. Resonant a.c. amplitude *B*_a.c._ versus fidelity *F* at *Φ*_a.c._ = π/2. Points show the mean of *n* = 5 independent measurements, where each measurement corresponds to the diamond being separately rehyperpolarized and run through the full pulse sequence. Error bars denote mean ± s.d. The solid line is a spline-fit guide to the eye. The right axis shows a normalized lifetime extension (>3,500-fold ${T}_{2}^{{\prime} }$ increase for 82.4 μT). Inset: time-domain profiles of the representative points in **b**: (i) no a.c. field, (ii) intermediate field *B*_a.c._ = 8.24 μT and (iii) *B*_a.c._ = 82.4 μT.

Lifetime enhancement (Fig. 1c(i)) also yields a change in the measured signal at every fixed time *t* compared with the case without an additional a.c. field and, hence, enables a means to sense the a.c. field. We now consider how this extension applies to the a.c. field characteristics (Fig. 2a(i)), ${B}_{{\rm{a}}.{\rm{c}}}(t)={B}_{{\rm{a}}.{\rm{c}}}\sin (2\pi {f}_{{\rm{a}}.{\rm{c}}}t+{\Phi }_{{\rm{a}}.{\rm{c}}})$, that is, its (1) phase *Φ*_a.c._, (2) amplitude *B*_a.c._ and (3) frequency *f*_a.c._.

To quantify the signal enhancement, we devise a fidelity metric that remains accurate even when the signal approaches the noise floor:

1 $$F=\frac{1}{{N}^{{\prime} }}\mathop{\sum }\limits_{i=1}^{{N}^{{\prime} }}\langle {I}^{x}({t}_{i})\rangle P({t}_{i}),$$

where *P*(*t*) represents the ideal DTC toggling response, alternating between ±1 as spins flip between the $\pm \widehat{{\bf{x}}}$ axes, and the normalized sum is carried out over all ${N}^{{\prime} }$ time points ${t}_{i}\in [{t}_{1},{t}_{{N}^{{\prime} }}]$; *F* yields the largest value when the DTC oscillations are the strongest and most stable. Formally, *F* corresponds to a weighted summation over the Fourier harmonics $\ell {f}_{res}$ ($\ell \in {{\mathbb{N}}}_{\, > \,0}$); when approaching the noise floor, it is more robust than the standard approach of estimating the PDTC response from only the period-doubling (that is, ${f}_{res}$) component. Lacking an analytical model for the DTC decay, the fidelity metric provides a simple, profile-agnostic measure of stability by integrating the observed magnetization. A more detailed understanding of the form of the DTC decay and its response to external magnetic field could provide a more optimized metric to improve the sensitivity of our approach.

Using this metric, Fig. 2a(ii) examines the impact of the a.c. field phase *Φ*_a.c._ on resonance ${f}_{{\rm{a}}.{\rm{c}}}={f}_{{\rm{r}}{\rm{e}}{\rm{s}}}$ and *γ*_*y*_ = π (blue data points). Maximum lifetime extension occurs at *Φ*_a.c._ = π/2, where the a.c. field peaks align with the centre of the *γ*_*y*_ pulses, as predicted theoretically (Supplementary Section 7). When the a.c. field nodes coincide with the *γ*_*y*_ pulses, there is a minimal effect on the PDTC lifetime. Optimal sensing, therefore, occurs when *Φ*_a.c._ = π/2. We observe a slight phase shift in the response from the expected maximum at *Φ*_a.c._ = π/2, due to unaccounted pulse transients when setting the a.c. field phase based on the pulse length (Fig. 2a(i)). This apparent shift arises because the actual pulse applied to the probe is slightly longer than the one generated by the arbitrary waveform transceiver^53^.

Additionally, the blue data points in Fig. 2a(ii) show the response to slight deviations from the small point, here *γ*_*y*_ = 0.98π. The data confirm the robustness of the PDTC order. In Supplementary Fig. 2, we display the entire experimentally mapped PDTC phase diagram for all *γ*_*y*_ values, demonstrating a large stable region around *γ*_*y*_ = π, independent of *t*.

Figure 2b studies the effect of the a.c. field strength *B*_a.c._, set at resonance with *Φ*_a.c._ = π/2. The fidelity profile shows a gradual increase, followed by a linear rise and eventually plateaus at higher field strengths when *B*_a.c._ becomes comparable with the Rabi field of the *θ*_*x*_ Floquet pulses. The right vertical axis shows the corresponding ${T}_{2}^{{\prime} }$ lifetimes; the maximum extension, corresponding to ${T}_{2}^{{\prime} }=21.3\,s$, is >3,000 fold. Here data are for *N* = 4; Supplementary Section 4A discusses the extensions obtained as a function of *N*. Figure 2b illustrates the time profiles of $\langle {I}^{x}\rangle$ for three cases: (i) no field *B*_a.c._ = 0, (ii) *B*_a.c._ = 8.24 μT and (iii) *B*_a.c._ = 82.4 μT. Figure 2b indicates that this can be used for sensing at appropriately chosen bias points. The sensitivity is determined by comparing the response of the fidelity *F* to perturbation in the a.c. field amplitude *B*_a.c._ (Fig. 2b), with the fluctuations in *F* obtained for multiple initializations at fixed *B*_a.c._ (Methods and Supplementary Section 4D). We obtain a sensitivity of 880 pT Hz^−1/2^ with an optimum bias field of *B*_a.c._ = 415 nT.

**Fig. 3:**
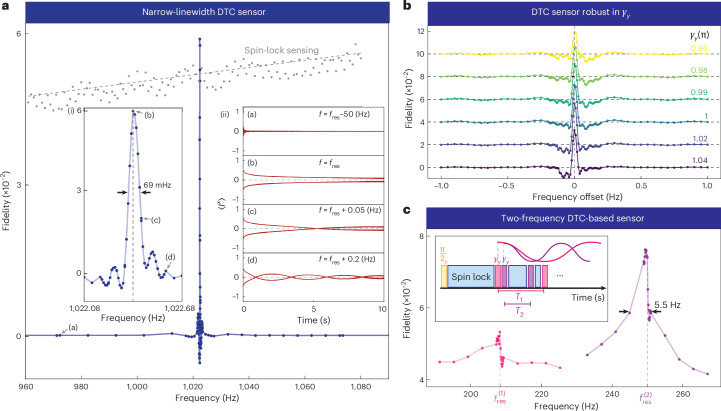
PDTC-based a.c. magnetic field sensing. **a**, Narrow-linewidth a.c. sensing: fidelity *F* (blue data points) is measured by sweeping the frequency *f*_a.c._ with *B*_a.c._ = 8.24 μT and *N* = 4, keeping other parameters consistent with those in Fig. 1c. A sharp increase in the PDTC lifetime (and hence, fidelity) occurs at the resonance condition ${f}_{AC}={f}_{res}$. By contrast, the spin-lock sensing scheme introduced in ref. ^54^ (grey data points) lacks frequency selectivity. (i) Zoomed-in view into the resonance feature, showing a narrow linewidth Δ*ℓ* ≈ 70 mHz, determined by ${({T}_{2}^{{\prime} })}^{-1}$. Points (b)–(d) are marked on the spectral wing. (ii) Time-domain PDTC profiles of $\langle {I}^{x}\rangle$ at points (a)–(d) in **a** and (i) at various offset frequencies from resonance. (a) Far off-resonance: fast signal decay, similar to bare PDTC case. (b) On resonance: substantially extended PDTC lifetime. (c) and (d) Slightly off-resonance, showing long lifetimes with beat pattern at frequency $\delta f={f}_{AC}-{f}_{res}$, resulting in ${T}_{2}^{{\prime} }$-limited a.c. sensing. Supplementary Section 4E discusses exploiting this for noise-rejected sensing: (π/2)_*y*_ and *θ*_*x*_ pulses (~51.5 μs); *γ*_*y*_ pulse (~103 μs); *τ* (~36 μs). **b**, Robustness of the resonance feature to deviations in *γ*_*y*_ kick angle, *γ*_*y*_ = π − *ϵ* (colour bar). The fidelity baselines for different *γ*_*y*_ kick angles were offset by 2 × 10^−2^ to prevent overlap. Data show that the spectral width Δ*ℓ* remains independent of *ϵ*. Similar experimental mapping of the PDTC phase diagram with respect to *γ*_*y*_ is shown in Supplementary Fig. 2. (π/2)_*y*_ and *θ*_*x*_ pulses (~52.25 μs); *τ* (~36 μs); *γ*_*y*_ pulse length scales linearly with its angle, with a π pulse (~104.5 μs). **c**, Tunable sensor profile for two-frequency sensing. Inset: sequence with two interspersed *γ*_*y*_ pulse blocks, leading to two resonance conditions: ${f}_{res}^{(1)}$ and ${f}_{res}^{(2)}$. *B*_a.c._ = 32.96 μT. Main panel: measured frequency response (similar to **a**), showing a two-tone response centred at 208 Hz and 250 Hz, with a narrow linewidth Δ*ℓ* ≈ 5.5 Hz. (π/2)_*y*_ and *θ*_*x*_ pulses (~50.25 μs); *γ*_*y*_ pulse (~100.5 μs). *t*_acq_ ≈ 13.6 μs for **c**.

A distinguishing feature of the a.c.-field-mediated lifetime extension is its strongly resonant nature. Figure 3a examines the fidelity *F* across a range of a.c. field frequencies *f*_a.c._ under identical conditions. Off-resonant frequencies have a negligible impact on the DTC lifetime, matching the bare PDTC (*B*_a.c._ = 0, *F* ≈ 0). By contrast, a notable lifetime increase is observed on resonance ${f}_{res}$ (Fig. 3a). A zoomed-in view (Fig. 3a(i)) reveals a narrow linewidth of Δ*f* ≈ 70 mHz set by the maximum integration time (${t}_{{N}^{{\prime} }}$ in equation (1)), positively correlated with the inverse of the PDTC lifetime ${({T}_{2}^{{\prime} })}^{-1}$. We also note a weak additional response at subharmonics, especially ${f}_{res}/2$, at large *B*_a.c._. This is discussed in Supplementary Section 4C (Supplementary Fig. 5).

The sharp ${f}_{res}$ response is further clarified using the representative points marked in Fig. 3a(i). The corresponding time-domain PDTC profiles are shown in Fig. 3a(ii). Far off-resonance (point (a)), the dynamics remain unaffected by the a.c. field. Exactly on resonance (point (b)), a notable lifetime increase is observed. Slightly off-resonance (points (c) and (d)), a distinctive beating in the fidelity *F* appears, reflecting the frequency offset $\delta f={f}_{{\rm{a}}.{\rm{c}}}-{f}_{{\rm{r}}{\rm{e}}{\rm{s}}}$. The integration of this beating pattern over time leads to the ${({t}_{N}^{{\prime} })}^{-1}$ linewidth. In sensing applications, this can enable a precise reconstruction of unknown signals within the narrow resonance band Δ*f* via a Fourier transform of the DTC temporal dynamics. The sensor bandwidth itself is determined by the shortest possible pulse lengths, and could span the 0.5–50-kHz range (Supplementary Fig. 1).

The dynamics of the tracked phase *ϕ* corresponding to Fig. 3a(ii) is presented in Supplementary Section 4B. The data (Supplementary Fig. 4) reveal intricate micromotion dynamics and demonstrate the ability to measure it for periods well beyond the 1/*e* lifetimes, exceeding 60,000 *γ* kicks, with high clarity.

We emphasize that the lifetime-limited linewidth is a feature of DTC-based sensing, distinguishing it from other methods such as magnetometry using spin-locked prethermal states introduced in ref. ^54^. Figure 3a (grey data points) illustrates the response of the spin-lock sensing scheme over the same frequency range in which we measured the DTC’s response. Unlike DTC-based sensing, the spin-lock sensing scheme can detect multiple frequencies without modifying the pulse sequence parameters, but as indicated by the data, it exhibits monotonically increasing response with frequency, lacking frequency selectivity. Even on resonance, the linewidth of the spin-lock sensing scheme can extend to several hundred hertz—at least four orders of magnitude broader—primarily dominated by interspin couplings, and largely independent of ${T}_{2}^{{\prime} }$. A detailed analysis of the spin-lock sensing data is provided in Supplementary Section 4H. By contrast, the narrow linewidth of the DTC sensing scheme enables tuning into specific fields that meet the resonance condition, effectively rejecting non-resonant fields (Supplementary Fig. 7). More broadly, compared with conventional quantum sensors based on electronic spins^55^, the two-tone cavity-interrogated nuclear PDTC allows single-shot, quasi-continuous sensing for $> 5{T}_{2\,}^{{\prime} \,}( > 100\,{\rm{s}})$ (Fig. 1c(ii) and Supplementary Fig. 4) without sensor reinitialization, with the resonant lifetime extension enhancing the sensor precision.

Another consequence of the PDTC order is that the narrow sensing linewidth remains highly robust to pulse errors *ϵ* in the *γ*_*y*_ pulses away from *γ*_*y*_ = π. This is shown in Fig. 3b with *γ*_*y*_ denoted by the colour bar. The sensor linewidth (zoomed-in view shown in Fig. 3a(i)) remains unaffected by these errors. Additionally, the system exhibits a remarkable tolerance to on-site disorder (Supplementary Section 7D) and fluctuations in the spin-lock *θ*_*x*_ drive, evidenced in the capacity of reliably applying >10^6^ *θ*_*x*_ pulses even with realistic imperfections (due to Rabi frequency heterogeneity) in these experiments.

The two-tone PDTC discussed so far (Fig. 1b(i)) hosts a single resonance frequency ${f}_{res}$, tunable via the sequence parameter *T*. However, it is possible to expand the number of resonance frequencies and adjust the DTC sensing spectrum by modifying the PDTC sequence. For instance, Fig. 3c(i) introduces a three-tone PDTC, establishing two resonance conditions at ${f}_{res}^{(1)}$ and ${f}_{res}^{(2)}$, achieved through two different interleaved periods for the *γ*_*y*_ pulses, interspersed with spin-lock *θ*_*x*_ pulses. The experimental response in Fig. 3c shows two distinct frequencies separated by ~42 Hz. We observe asymmetric spectra with a stronger *x* component when the period *T* is decreased, leading to a more frequent overlap between the a.c. field antinodes and the *γ*_*y*_ pulses. This increased overlap enhances the *x*-component response (Supplementary Section 6B). The two-frequency linewidths, around 5 Hz, remain appreciably narrower than the sensor’s linewidth without DTC order (~223 Hz)^54^, although single-frequency linewidths (Fig. 3a) are narrower due to longer ${T}_{2}^{{\prime} }$ lifetimes in the two-tone case.

### a.c.-field-mediated lifetime extension for single-tone PDTC

Lifetime enhancement from a.c. field-mediated finite energy density applies broadly to all *U*(1) PDTCs, not just the two-tone PDTC. To demonstrate this, we consider a conventional single-tone DTC that alternates between the $+\widehat{{\bf{z}}}$ and $-\widehat{{\bf{z}}}$ states on the Bloch sphere. This approach is widely used across platforms, including superconducting qubits^8,11^, cold atoms^56^ and nuclear magnetic resonance (NMR)^4,5,12^. Unlike the two-tone DTC, which enables non-destructive inductive read-out in the $\widehat{{\bf{x}}}$–$\widehat{{\bf{y}}}$ plane to monitor the decay dynamics in a single shot, the single-tone DTC requires restarting the experiment for each data point.

**Fig. 4:**
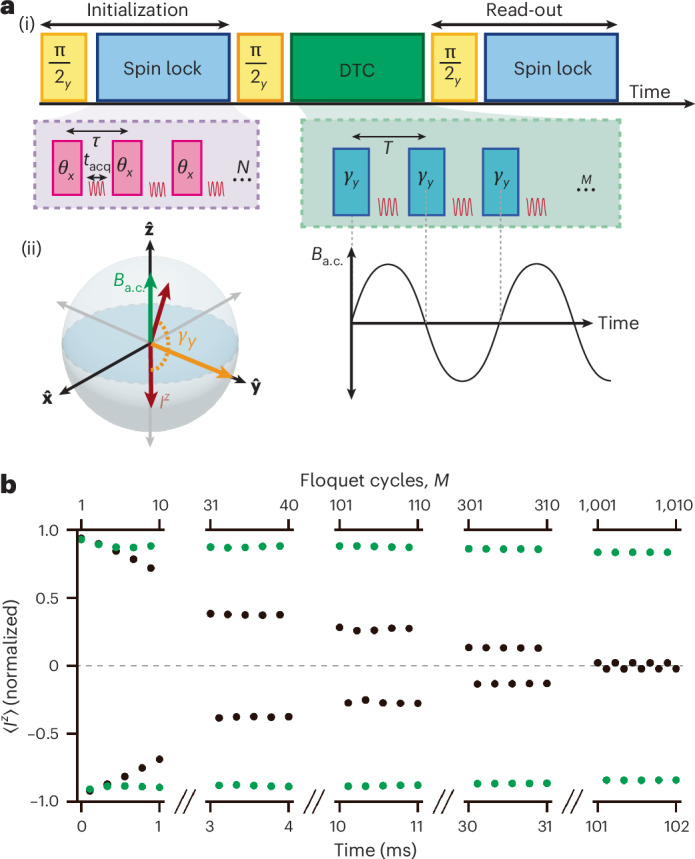
Single-axis PDTC extension under a.c. field. **a**, (i) Protocol: PDTC sequence (green) consists of a train of *M* 100.5-μs *γ*_*y*_ = π pulses along $\widehat{{\bf{y}}}$, spaced by 300 μs, flipping the spins between $+\widehat{{\bf{z}}}$ and $-\widehat{{\bf{z}}}$, robust to deviations from *γ*_*y*_ = π (ref. ^10^). Spin-lock trains before and after the DTC sequence read the initial and final spin population $\langle {I}_{z}\rangle$, enhancing the signal-to-noise ratio. (ii) Spin motion on a Bloch sphere. PDTC oscillates along the same axis as the applied a.c. field ($\widehat{{\bf{z}}}$), unlike the two-tone case in which the axes are orthogonal. **b**, PDTC lifetime extension. Black data points: normalized single-axis PDTC signal $\langle {I}_{z}\rangle$ without an a.c. field, decaying with ${T}_{2}^{{\prime} }=0.01\,s$ or *M* = 35 flips. Green data points: DTC under a resonant a.c. field with *B*_a.c._ = 1 μ*T*, showing a substantially extended lifetime of ${T}_{2}^{{\prime} }=20\,s$, or *M* = 70,000 spin flips—a 2,000-fold increase. Each data point requires restarting the experiment.

The sequence is shown in Fig. 4a(i), and consists of *M* spin-flip *γ*_*y*_ pulses along $\widehat{{\bf{y}}}$ (Fig. 4a(ii) describes this using a schematic). We utilize the exact *U*(1) symmetry of the dipole–dipole Hamiltonian *H*_dd_, which conserves $\widehat{{\bf{z}}}$ magnetization: $[{H}_{dd},{I}^{z}]=0$. For efficient read-out, spins are tipped onto the $\widehat{{\bf{x}}}\,-\,\widehat{{\bf{y}}}$ plane and spin locked using a train of *θ*_*x*_ pulses along $\widehat{{\bf{x}}}$. Unlike the two-tone case shown in Fig. 1, the data here are collected point by point for different values of *M*.

The results are shown in Fig. 4b. Without an a.c. field (black data points), we observe robust period-doubling dynamics with a 1/*e* decay time $T{\prime} \approx 0.01\,s$ and $M=40\,\widehat{{\bf{z}}}\leftrightarrow -\widehat{{\bf{z}}}$ flips. With a resonant a.c. field of *B*_a.c._ = 40 μT (green data points), the lifetime is markedly prolonged, extended by more than three orders of magnitude to *M* = 70,000 and ${T}_{1}^{{\prime} }=20\,s$. The phase response to *Φ*_a.c._ is opposite to that shown in Fig. 2a(ii), as the a.c. field direction aligns with the PDTC oscillation axis; here it is the maximum near *Φ*_a.c._ = 0 (Supplementary Fig. 11). Although the quasi-continuous measurement from the two-tone drive is more suitable for sensing applications, the data in Fig. 4b demonstrate that this lifetime extension mechanism applies broadly to *U*(1) PDTCs.

## Discussion

This work introduces several novel features. The key conceptual result is the resonant extension of the PDTC lifetime via an a.c. field, exponentially suppressing the heating rate relative to the driving period *T* (Supplementary Section 6). As shown in Fig. 2b, we extend the PDTC 1/*e* lifetime to ${T}_{2}^{{\prime} } > 20\,s$ and *M* = 40,000 spin-flipping Floquet cycles. Compared with previous work^1–3,7,10–12^, this sets a new record for both parameters, representing over two orders of magnitude improvement in the total number of DTC spin flips. Supplementary Table 1 provides a detailed comparison, highlighting the remarkably increased DTC lifetime in our case, despite similar interaction strengths and pulsing rates to previous works.

The methodology introduced here is not limited to nuclear spins and can be applied to a wide range of quantum and classical systems. We anticipate immediate applications to cavity-interrogated NV centres^57^ for quantum sensing in the range of 1 MHz to 1 GHz (ref. ^58^). The underlying principles themselves are also applicable to superconducting qubits^8,11^, cold atoms^56^ and ions^3^.

Crucially, sensing based on many-body DTC order naturally tolerates strong interspin couplings, leading to a lifetime-limited linewidth ($\sim ({T}_{2}^{{\prime} })$) rather than being dominated by couplings (~*J*^−1^). This enables sensing at high sensor densities, extending beyond the conventional regime of dilute, non-interacting sensors^42^; all else being identical, this can yield substantial sensitivity improvements^59^. Moreover, sensing here inherits the robustness of PDTC order against pulse sequence errors and sample inhomogeneities; we demonstrate this in Supplementary Section 7D, showing the resilience of a.c. sensing to strong on-site disorder. Finally, this approach does not require the preparation of strongly entangled states or fine tuning to a critical point. All these factors indicate broad applicability to a wide range of systems.

## Methods

### Materials

The sample used in this work is a single-crystal diamond measuring 3.4 × 3.2 × 2.1 mm^3^, containing a natural abundance of ^13^C nuclei and NV centres at ~1-ppm concentration. This same sample has been characterized in prior studies^10,46^, allowing for direct comparisons with the lifetime extensions observed. The sample is oriented parallel to the **B**_pol_ magnetic field, ensuring the simultaneous hyperpolarization of the four NV-centre axes, and experiments are carried out underwater to provide more uniform illumination and aid in thermal management^60^.

### Experimental setup

Instrumentation for hyperpolarization and ^13^C read-out follows previous works, with detailed descriptions available in those studies^10,45–47,54^. Data here are taken at room temperature and *B*_0_ = 7 T. Polarization is carried out at a low field centre (*B*_pol_ ≈ 38 mT) located below the magnet, driven by optically excited NV centres and chirped microwave excitation. The polarization mechanism involves successive Landau–Zener anticrossings in the rotating frame^61,62^. Sample shuttling to the *B*_0_ high field occurs in under 1 s, with an NMR saddle coil used for inductive read-out of the ^13^C precession signal.

^13^C interrogation is performed using a custom-built NMR spectrometer based on a high-speed arbitrary waveform transceiver (Proteus P9484M)^47,63^. The arbitrary waveform transceiver device generates NMR pulses and digitizes the nuclear precession directly at the Larmor frequency, eliminating insertion losses typically encountered with intermediate frequencies. The device’s high memory capacity (16 GB) and large sampling rate (up to 2.7 GS s^−1^) enable a continuous interrogation of ^13^C spin precession in windows between pulses. In typical experiments, we apply 0.2–1 million pulses with a read-out window of ~*f* ≈ 13.6 μs. The entire Larmor precession can be sampled every 0.74 ns and mixed with an onboard numerically controlled oscillator at the Larmor frequency, allowing us to track both amplitude and phase of the spins directly in the rotating frame^47,52^.

The receive chain amplifies the signal using low-noise preamplifiers (Advanced Receiver Research P75VDG and Pasternack PE15A1011), whereas the NMR pulse generation is via a Herley TWT amplifier through a Techmag transcoupler. For a.c. magnetometry, the spins are exposed to a weak magnetic field applied via a secondary coil, with the field parallel to $\widehat{{\bf{z}}}$ and positioned within the NMR probe. The field is applied via a Tektronix source, amplified by a Techron 7224 amplifier, and the field strength is calibrated through the voltage drop across a high-power 250-W 4-Ω resistor.

### Classification of temporal order

To place our work in the broader context of temporal order in closed systems, let us recall the different ways in which temporal order can be realized. In closed periodically driven systems, so far, three mechanisms are known to realize temporal order, namely, (1) many-body localized DTCs, (2) PDTCs and (3) *U*(1) PDTCs. The many-body localized DTCs require stable many-body localization^64,65^, which may only exist in one-dimensional short-range quantum systems in the presence of strong disorder^66^; under these conditions, the emerging spatiotemporal eigenstate order^15,67^ is entirely robust out to infinite times^68^. By contrast, the PDTCs require the existence of (pre)-thermal order, that is, a low-temperature symmetry-breaking state, namely, by the Mermin–Wagner theorem only possible in two and higher dimensions. The resulting prethermal spatiotemporal order has a finite lifetime determined by Floquet heating, and thus, exponentially suppressed in the driving period for short-range interacting systems^23,24^. Finally, *U*(1) PDTCs only require a (quasi-)conserved *U*(1) symmetry and an initial state that breaks this symmetry, irrespective of the effective temperature of this state. However, melting of the prethermal temporal order is dominated by symmetry-breaking perturbations, which, in the case of an emergent symmetry, only lead to the power-law suppression of heating in the driving frequency.

Our work focuses on the last class, the *U*(1) PDTCs, demonstrating that the lifetime of temporal order can be exponentially enhanced by coupling the system to the order parameter. Thus, the a.c.-enriched *U*(1) PDTCs effectively mimic the behaviour of PDTCs in terms of lifetime. We emphasize that adding the a.c. field to the *U*(1) PDTC is not sufficient to realize a thermally ordered state required for the PDTC.

### Sensitivity calculation

The sensitivity to a.c. magnetic fields based on the described effect of DTC extension is calculated by

M1 $$\mathrm{Se}{\rm{n}}{\rm{s}}{\rm{i}}{\rm{t}}{\rm{i}}{\rm{v}}{\rm{i}}{\rm{t}}{\rm{y}}=\frac{{\sigma }_{F}}{\partial F/\partial {B}_{{\rm{a}}.{\rm{c}}}}\sqrt{{t}_{\mathrm{int}}},$$

that is, the response of the signal metric (here fidelity *F*) to perturbation in the field strength *B*_a.c._ is compared with the noise *σ*_*F*_ in the metric at fixed field strength, normalized for the noise reduction achievable by integrating the signal over the measurement time *t*_int_. Sensitivity is determined via a scan of the amplitude *B*_a.c._. For each chosen amplitude, the DTC experiment is repeated several times. The fluctuation *σ*_*F*_ in the fidelity metric is determined by the standard deviation amongst trials at a fixed amplitude *B*_a.c._. The mean fidelity for trials at each amplitude is used in calculating the centred difference between adjacent amplitude points *B*_a.c._ to estimate the response ∂*F*/∂*B*_a.c._. As shown in Fig. 2b, the amplitude dependence of the DTC coherence extension is nonlinear; the response ∂*F*/∂*B*_a.c._ increases with *B*_a.c._ up to ~8 μT. This could suggest that the sensor should be operated with a bias a.c. field near 8 μT for best performance. However, the fluctuations in fidelity are also found to grow with *B*_a.c._, even more steeply than the response ∂*F*/∂*B*_a.c._. Thus, the best sensitivity is actually obtained for a small bias of 415 nT. Supplementary Fig. 6 shows the calculated sensitivity as a function of bias. The cause of the sharp rise in *σ*_*F*_ with an applied field is unclear; this could be due to an increased instability of the a.c. source itself or due to the increased susceptibility of the DTC to drifts in the instrument as a result of the bias field. The sensitivity and opportunities to improve it are further discussed in Supplementary Section 4D.

### Floquet engineering of finite energy density

As discussed below, in both single-tone and two-tone DTC, the a.c.-induced signal enhancement is the result of coupling the system to the DTC order parameter via the added a.c. field. The complete derivation can be found in the Supplementary Information.

#### Single-tone DTC

For the single-tone DTC, the order parameter corresponds to the $\widehat{{\bf{z}}}$ magnetization (〈*I*^*z*^〉) flipping sign every period *T*, that is, ${{\mathcal{O}}}_{1DTC}(\ell T)=\langle {(-1)}^{\ell }{I}^{z}\rangle$, thereby oscillating with a period 2*T*. Thus, naturally, an a.c. field in the $\widehat{{\bf{z}}}$ direction couples to the order parameter such that the system every full DTC period (2*ℓT*) is effectively described by *H*_a.c.,eff_ = *H*_dd_ + *B*_eff_*I*^*z*^, with the effective field *B*_eff_ proportional to the a.c. amplitude, *B*_eff_ ∝ *B*_a.c._.

#### Two-tone DTC

For the two-tone DTC, a key difference is that the order parameter oscillates in between $\widehat{{\bf{x}}}$ and $-\widehat{{\bf{x}}}$, ${{\mathcal{O}}}_{2DTC}(\ell T)=\langle {(-1)}^{\ell }{I}^{x}\rangle$, orthogonal to the a.c. field. However, crucially, the $\widehat{{\bf{y}}}$ pulses implementing the DTC sequence are of a finite time duration *τ*_*y*_, that is, a finite a.c. field is present also during the application of the $\widehat{{\bf{y}}}$ pulses. Indeed, one can show (Supplementary Section 6B) that for the *γ*_*y*_ = π pulses, applying the *I*^*y*^ field and *I*^*z*^ simultaneously corresponds to applying an *I*^*x*^ and an *I*^*y*^ field separately, that is,

$${{\rm{e}}}^{-{\rm{i}}({\gamma }_{y}{I}^{y}+{(-1)}^{n}{B}_{n,z}{\tau }_{y}{I}^{z})}\approx {{\rm{e}}}^{-{\rm{i}}{\gamma }_{y}{I}^{y}}{{\rm{e}}}^{-{\rm{i}}\frac{{(-1)}^{n}{B}_{n,z}{\tau }_{y}}{{\gamma }_{y}}{I}^{x}},$$

up to an error scaling as $O({({B}_{z}{\tau }_{y}/{\gamma }_{y})}^{2})$, where *n* labels the $\widehat{{\bf{y}}}$ pulse and ${B}_{z,n}=| {\int }^{{\tau }_{y}}{B}_{{\rm{a}}.{\rm{c}}}(t){\rm{d}}t/{\tau }_{y}|$ is the amplitude of the a.c. field accumulated during the *n*th $\widehat{{\bf{y}}}$ pulse. Therefore, indeed, the $\widehat{{\bf{z}}}$ a.c. field effectively induces an $\widehat{{\bf{x}}}$ a.c. field. In particular, this induced a.c. field is the strongest if the minima and maxima of the a.c. field align with the $\widehat{{\bf{y}}}$ pulses, in agreement with the experimental results shown in Fig. 2; Figs. 2 and 3 and Supplementary Section 7 provide a derivation of the other effects. Thus, similar to the single-tone case, the system in the presence of an a.c. field is effectively described by *H*_a.c.__,eff_ = *H*_eff_ + *B*_eff_*I*^*x*^ after every full DTC period (2*ℓT*), for the effective interacting Hamiltonian ${H}_{e\mathrm{ff}}\approx {\sum }_{k < l}{J}_{kl}({I}_{k}^{x}{I}_{l}^{x}-{{\bf{I}}}_{k}\cdot {{\bf{I}}}_{l})$ and effective field *B*_eff_ ∝ *B*_a.c._; Supplementary Section 6B provides the derivation.

#### Lifetime enhancement via ETH

In both cases, the system over a full DTC cycle (2*T*) is effectively described by an interaction part and an emergent static field with strength proportional to the a.c. field strength, *H*_a.c.,eff_ = *H*_int_ + *B*_eff_*I*^*α*^, where *α* = *z*, *x* for single- and two-tone drives, respectively. Note that for small imperfections in the drive *γ*_*y*_ ≠ π, the ‘interaction’ terms *H*_int_ will break the *U*(1) conservation law, [*H*_int_, *I*^*α*^] ≠ 0. Therefore, the only conserved quantity is the quasi-conserved energy, 〈*H*_a.c.,eff_〉, in the prethermal plateau. Thus, the ETH^48–51^ predicts that a generic system starting in an initial state $| {\psi }_{0}\rangle$ will relax at ‘late’ times to a state $| {\psi }_{\infty }\rangle$, which is indistinguishable from a thermal state ${\rho }_{{\mathcal{T}}}\propto {e}^{-{H}_{{\rm{a}}.{\rm{c}},{\rm{e}}{\rm{f}}{\rm{f}}}/{\mathcal{T}}}$, with temperature ${\mathcal{T}}$ determined by energy conservation. This means, for spatially local observables *A*, like energy or magnetization, the expectation value in the late-time state is equal to the thermal expectation value: ${\langle A\rangle }_{{\psi }_{\infty }}={\langle A\rangle }_{{\rho }_{{\mathcal{T}}}}$. The same holds for the initial mixed state *ρ*_0_.

Importantly, in the absence of an a.c. field, the energy of the initial state ${\rho }_{0} \sim {\mathbb{1}}+\mu {I}^{\alpha }$ for both single- and two-tone DTC vanishes (${\langle {H}_{int}\rangle }_{{\rho }_{0}}=0$), thereby leading to (pre)thermalization to a featureless infinite-temperature state ${\rho }_{{\mathcal{T}}=\infty }\propto {\mathbb{1}}$. By contrast, in the presence of an a.c. field, the initial energy becomes finite, ${\langle {H}_{{\rm{e}}\mathrm{ff},{\rm{a}}.{\rm{c}}}\rangle }_{{\rho }_{0}}\propto \mu {B}_{{\rm{a}}.{\rm{c}}}$; thus, the system prethermalizes to a finite-temperature state ${\rho }_{{\mathcal{T}}\ne \infty }\propto {e}^{-{H}_{{\rm{a}}.{\rm{c}},{\rm{e}}{\rm{f}}{\rm{f}}}/{\mathcal{T}}}$, with finite magnetization ${\langle {I}^{\alpha }\rangle }_{{\rho }_{{\mathcal{T}}\ne \infty }}\ne 0$. This prethermal value persists until the ultimate melting of the prethermal plateau, which is exponentially suppressed in the period *T*, ${\varGamma }_{{\rm{e}}}^{{\rm{a}}.{\rm{c}}}\propto \exp (-c/JT)$ for some constant *c*. Thus, the lifetime can be exponentially enhanced by introducing an additional a.c. field (Supplementary Section 6).

### Overview of requirements for a.c.-induced signal enhancement

Although the above analysis focused on dipole-coupled nuclear spins in diamond, the mechanism applies more generally. In a nutshell, the key ingredients are (1) (emergent) symmetry-protected period-doubling response; (2) the system should (pre)thermalize in agreement with ETH; (3) a high-temperature initial state such that the lifetime is limited by symmetry-breaking terms; and (4) the ability to (effectively) couple the system to the DTC order parameter, for example, via Floquet engineering. Note that since the DTC order parameter itself oscillates in time with period 2*T*, inducing a coupling to the order parameter necessarily requires adding an additional time-varying (a.c.) field. Then, by adding this time-varying field, one can exponentially extend the lifetime of the PDTC order using the procedure presented above.

## Online content

Any methods, additional references, Nature Portfolio reporting summaries, source data, extended data, supplementary information, acknowledgements, peer review information; details of author contributions and competing interests; and statements of data and code availability are available at 10.1038/s41567-025-03163-6.

## Supplementary information

Supplementary Information Supplementary Figs. 1–18, Table 1 and Discussion.

## Data Availability

All data supporting the findings of this study are available via Zenodo at 10.5281/zenodo.17744537(ref. ^69^).

## Code availability

All code used in this study is available via Zenodo at 10.5281/zenodo.17744537 (ref. ^69^).
