# Supplementary material for: Sensing with discrete time crystals
Source: Nat Phys. 2026 Feb 23;22(3):367–73. doi: 10.1038/s41567-025-03163-6 (PMC13046468; doi:10.1038/s41567-025-03163-6)
Supplement: Supplementary file 1 — Supplementary Figs. 1–18, Table 1 and Discussion. [file 41567_2025_3163_MOESM1_ESM.pdf]

nature physics

Supplementary information https://doi.org/10.1038/s41567-025-03163-6

Sensing with discrete time crystals

In the format provided by the
authors and unedited

Supplemental Information:
Sensing with discrete time crystals

CONTENTS

S1. Lifetime comparison 1

S2. Sensor comparison 1

S3. Phase diagram mapping: action of AC field 1

S4. Extended data 3
- A. Lifetime extension as a function of N 3
- B. Phase measurement for off-resonance fields 3
- C. Sub-harmonic response at large B_{AC} 4
- D. Sensitivity 4
- E. Noise rejection of the DTC sensor 4
- F. Estimating magnetic field from the $\hat{\mathbf{z}}$-coil 5
- G. Three-tone drive 5
- H. Spin-lock sensing 6
- I. Phase-dependence of single-tone DTC 7

S5. Numerical Algorithm 7

S6. Floquet engineering finite energy density 9
- A. One-tone prethermal discrete time crystal 10
- B. Two-tone prethermal discrete time crystal 10
- C. Summary of AC-induced signal enhancement 12
- D. AC vs DC field comparison 14

S7. Characteristics of AC enhanced signal 14
- A. Amplitude 15
- B. Phase 15
- C. Frequency 15
- D. Noise resilience 15
- E. Dependence on spin density 15

S8. Sensing AC field with an unknown phase 18

S9. Effect of AC field during γ_y pulses 19

References 20

S1. LIFETIME COMPARISON

In Supplementary Table 1 we compare the DTC lifetimes achieved in this work with other lifetimes in the literature, including other platforms and DTC orders. Note that, in principle the MBL DTC realized using superconducting qubits [1] has an infinite lifetime, however, the DTC lifetime is limited by the coherence of the device. Our AC-enhanced $U(1)$ PDTC exceeds previous experimental DTC realizations by at least one order of magnitude considering the total number of Floquet pulses applied, here $40,000$.

S2. SENSOR COMPARISON

To compare with other magnetometers, we illustrate the demonstrated and prospective sensitivity of the ^{13}C DTC sensor along with various others, as a function of the magnetic field frequencies they probe, in Supplementary fig 1. Sensor numbers 1-26 correspond to Refs. [10–35]. Although sensors 3, 4, 20 and 25 do achieve better sensitivity than we have yet demonstrated at frequencies between 1 kHz and 10 kHz, these are significantly larger sensors at 100 mm^3, 39000 mm^3, 5300 mm^3, and 1257 mm^3 respectively compared to the sensing volume 22.7 mm^3 of our diamond sample. Larger sensing volume necessitates greater standoffs from local field sources, which offsets sensitivity advantages. Larger sensors are also more vulnerable to performance degradation due to ambient field gradients, and are less apt for mapping inhomogeneous fields at high resolution.

Sensors 16 and 26 are the only ones identified which can sense fields between 1 kHz and 20 kHz with higher sensitivity in a smaller volume than ours. For practical applications, we note that certain additional properties might advantage one sensor over another among this small set. For example, our sensor is demonstrated at a high bias field of 7 T, compared to the bias fields of 0.178 T and 0.7 mT for 26 and 16 respectively. This is advantageous for instance in NMR applications, since the higher bias strengthens the field source from nuclear spins (offsetting discrepancy in the magnetometer sensitivity). It also increases chemical shift, enabling better resolution of NMR spectra. We also note that robustness to environmental conditions may differ among these sensors. For instance, vibrational noise can pollute the magnetic field signal in the case of the ferrimagnetic oscillator [35], while we do not anticipate a direct imprint on DTC stability from vibrations.

S3. PHASE DIAGRAM MAPPING: ACTION OF AC FIELD

To demonstrate the efficacy of the applied AC field in extending the DTC phase, we systematically mapped the DTC phase diagram using the technique from Ref. [9]. Unlike other DTC experiments, which record the trace point by point after each Floquet pulse, our approach captures the entire trace in a single shot. This enables mapping the phase diagram by measuring multiple slices as a function of the γ_y-pulse angle.

Supplementary fig 2A shows the DTC phase diagram without an AC field, similar to Beatrez et al. [9]. In this experiment, we use N=16, meaning γ_y pulses are applied in rapid succession, separated by only 16 spin-lock pulses. This smaller N results in a faster DTC decay compared to Ref. [9].

Data in Supplementary fig 2A is presented in both linear and log scales. The y-axis represents the total number of Floquet cycles, indicated by the number of γ_y-pulses applied, while the x-axis represents the γ_y flip-angle. Colors indicate the obtained signal, with alternating green and magenta stripes

	Platform	DTC type	Mean interactions	Period	Spin lifetime	Floquet cycles
Mi et.al. 2022 [1]	Superconducting qubits	MBL	–	–	≈ 6.4 μs	≈ 50–100
Zhang et.al. 2017 [2]	Trapped Ions	prethermal	≈ 0.04–0.25 kHz	≈ 74 μs	≈ 7 ms	≈ 100
Kyprianidis et.al. 2021 [3]	Trapped Ions	prethermal	≈ 0.33 kHz	≈ 280–500 μs	≈ 12–19 ms	≈ 50–100
Choi et.al. 2017 [4]	NV centers	$U(1)$	≈ 105 kHz	≈ 92–998 ns	≈ 60 μs	≈ 50
Rovny et.al. 2018 [5]	ADP ^{31}P	$U(1)$	≈ 508 Hz	10 μs–1 s	–	≈ 50
Pal et.al. 2018 [6]	Acetonitrile, TMP, TTSS ^{1}H	$U(1)$	≈ 2.5–136 Hz	–	–	≈ 20–60
Stasiuk et.al. 2023 [7]	Fluorapatite ^{19}F	$U(1)$	≈ 0 – 5.2 kHz	≈ 120 μs	≈ 9.3 ms	≈ 80
Randall et.al. 2021 [8]	Diamond ^{13}C	prethermal/MBL	≈ 6.7 Hz	≈ 5 ms	≈ 2.5 s	≈ 500
Beatrez et.al. 2022 [9]	Diamond ^{13}C	$U(1)$	≈ 0.66 kHz	≈ 5–50 ms	≈ 14 s	≈ 500
This work	Diamond ^{13}C	$U(1)$	≈ 0.66 kHz	≈ 1.5 ms	≈ 21.29 s	≈ 44, 200

Supplementary Table 1. **Comparison of DTC lifetimes in other experiments and platforms.** We compare the lifetimes of other DTC experiments in the literature with the lifetimes achieved in this work.

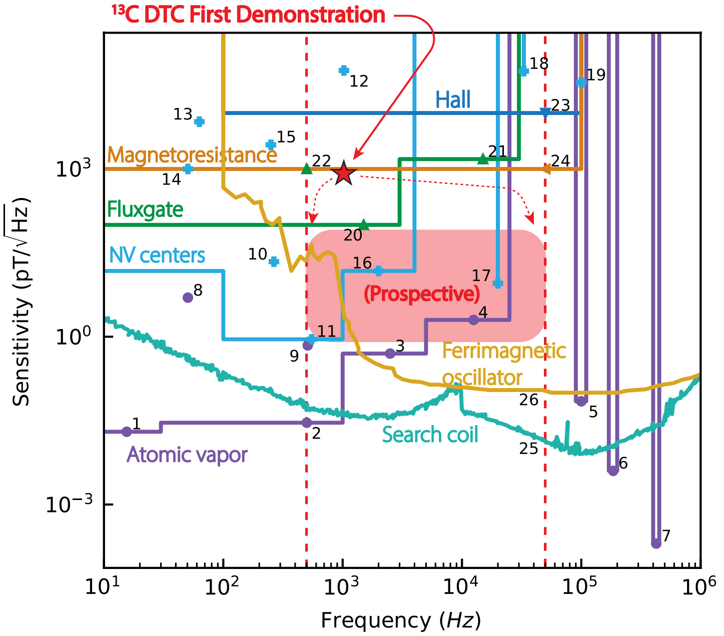

(1) Zhang et al. Sci. Adv. 6, eaba8792 (2020). **(2)** Groeger et al. Eur. Phys. J. D 38, 239-247 (2006). **(3)** Ijsselsteijn et al. Rev. Sci. Instrum. 83, 113106 (2012). **(4)** Kurian et al. Phys. Rev. Applied 19, 054040 (2023). **(5)** Lucivero et al. Rev. Sci. Instrum. 85, 11 (2014). **(6)** Schultze et al. Sensors 17 (2017). **(7)** Alem and Saner. Phys. Rev. A87, 013413 (2013). **(8)** Schwindt et al. Appl. Phys. Lett. 90, (2007). **(9)** Li et al. Meas. 190 (2022). **(10)** Chtzidrosos et al. Phys. Rev. Applied 8, 044019 (2017). **(11)** Fescenko et al. Phys. Rev. Res. 2, 023394 (2020). **(12)** Kuwahata et al. Sci. Rep. 10, 2483 (2020). **(13)** Webb et al. Appl. Phys. Lett. 114, 231103 (2019). **(14)** Clevenson et al. Appl. Phys. Lett. 112, 160802 (2014). **(15)** Jensen et al. Phys. Rev. Lett. 112, 160802 (2014). **(16)** Barry et al. Proc. Natl. Acad. Sci. U. S. A. 113, 49 (2016). **(17)** Wolf et al. Phys. Rev. X 5, 041001 (2015). **(18)** Maletinsky et al. Nature Nanotech 7, 320-324 (2012). **(19)** Yahata et al. Appl. Phys. Lett. 114, 022404 (2019). **(19)** Lu et al. Sensors 14 (2014). **(20)** Bartington Instruments, UK. **(21)** Texas Instruments, USA. **(22)** Metrolab Technology SA, Switzerland. **(23)** Haned and Missous, Sensors and Actuators A 102, 3 (2003). **(24)** Díaz-Michelena, Sensors 9, 4 (2009). **(25)** Coillot et al. IEEE Sensors 10, 2 (2009). **(26)** Barry et al. Phys. Rev. Applied 19, 044044 (2023).

Supplementary fig 1. **Comparison of magnetometer sensitivity** as a function of frequency. Red dashed lines indicate the frequency regime in which the sensor presented here is expected to operate. The red shaded area indicates prospected sensitivity with improvements described in SI Sec. S2. Contours indicate the best sensitivity the authors have identified at a given frequency for a given type of magnetometer. For the NV magnetometers labeled 17-19, no bandwidth was identified, hence only vertical contours are ascribed to these. The sensor concept demonstrated in this paper is best suited for sensing of ~kHz frequencies, a range at which other existing sensor technologies lose substantial sensitivity. Comparison to the most sensitive magnetometers operating at similar frequencies is made in SI Sec. S2. Note that superconducting quantum interference devices (SQUIDs) and certain atomic magnetometers are not included here. While these can offer excellent sensitivity, operational constraints limit their applications. Namely, some atomic magnetometers can only function in a shielded, low-field environment, and SQUIDs require cryogenic cooling.

representing the period-doubling response as the spins flip between $+\hat{\mathbf{x}}$ and $-\hat{\mathbf{x}}$. Two stable points are evident, centered at γ_y=0 and γ_y=π. White regions indicate where the spins undergo complete decoherence.

In the conventional DTC case, signal decay occurs in ~100 Floquet cycles for N=16, similar to Ref. [9] where N=32. Supplementary fig 2B contrasts this with the effect of a B_{AC}=82 μT AC magnetic field, showing a significantly extended stability region near γ_y=π.

Data is presented with a split axis for clarity, as the lifetime extension is over 200 times larger compared to without the AC field, sustaining over 10,000 γ-kicks. We observe a very stable period-doubling spin-flipping response. Importantly, the stability *"dome"* around γ_y=π is retained, reflecting the characteristic DTC behavior.

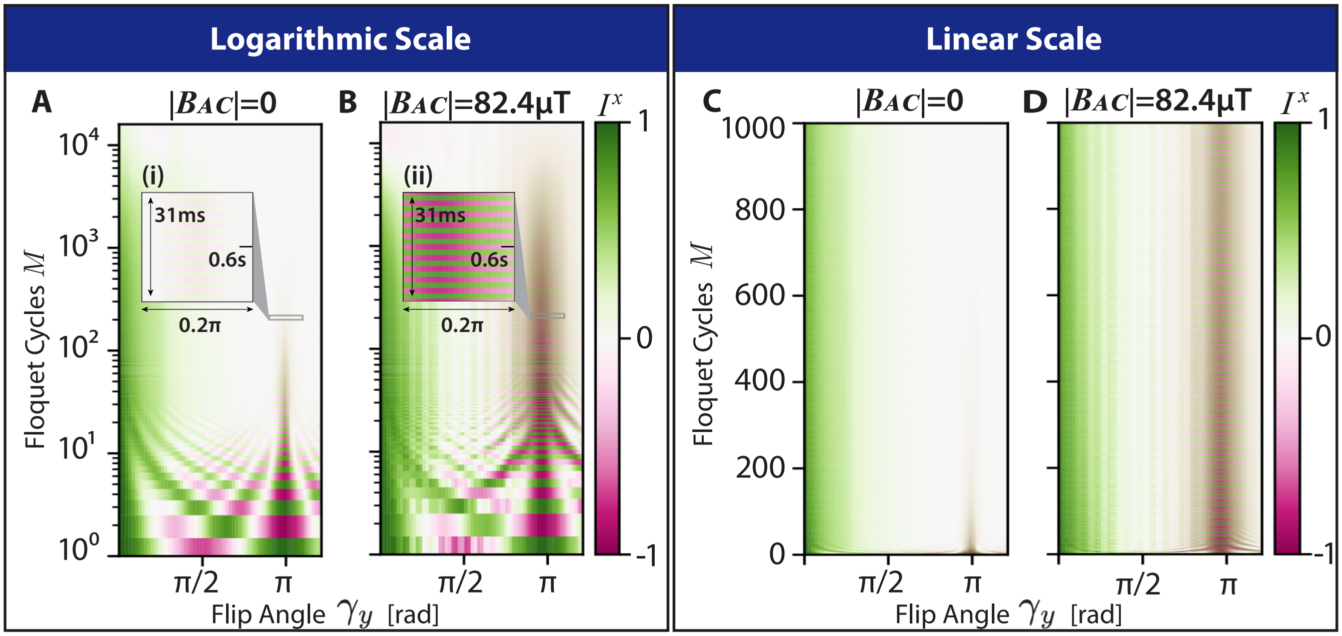

Supplementary fig 2. **Phase diagram mapping of PDTC revealing origin of lifetime extension**. Panels show mapped PDTC phase diagram for 100 γ_y-flip angles (vertical slices) in range $[0, 1.3\pi]$. In this experiment, pulse angle is assumed to be proportional to pulse length, with $\gamma_y = \pi$ corresponding to a pulse length of 103.5 μs. The y-axis represents number of Floquet cycles (γ_y kicks), while right axis shows total absolute time. Color bars represent $\langle I^x \rangle$ signal intensity. (A) Data on logarithmic scale without AC field. There is a characteristic stability dome around PDTC stable points at γ_y=$\{0, \pi\}$, as described in Ref. [9]. Without AC field, the DTC phase dissipates rapidly (shown for N=16). (B) With B_{AC}=82μT, the DTC phase lasts significantly longer, extending beyond 12,000 cycles, an extension factor over 200. Data reveals that with B_{AC}, the characteristic sharpening of the dome with increasing Floquet cycles is absent. Instead, there is a stability region around γ_y that is independent of γ_y and depends only on the AC field strength, matching theoretical expectations. (C-D) Right panels show same data on linear scale. Extension in DTC lifetime is evident. Brown color [not present on colorbar] is an optical illusion due to fast alternating green and magenta cycles on a log scale.

S4. EXTENDED DATA

A. Lifetime extension as a function of N

In the two-tone driving scheme, the number of pulses N effectively sets the timescale for prethermalization. Consequently, one expects that the extension of the PDTC lifetime under the AC field depends on the total number of spin-lock pulses employed. We note that in previous work demonstrating a long-lived PDTC (T_2'=4.3 s) and mapping its phase diagram, N was chosen to be a large number (N=300).

Supplementary fig 3 examines the effect of lifetime extension as a function of N. The data show that the lifetime extension is most dramatic for small N and appears to saturate for N>100. We rationalize this as follows: as N gets larger, the period T lengthens, shifting away from the high-frequency regime. This inhibits the spins to prethermalize into finite energy density states under the combined AC field and PDTC two-tone driving, ultimately preventing lifetime extension.

The small N regime, where PDTC extension is greatest (Supplementary fig 3), is also particularly important for sensing of higher-frequency RF AC fields with improved sensitivity.

B. Phase measurement for off-resonance fields

In Fig. 3A(ii) of the main paper, we show the polarization component $\langle I^x \rangle$ as a function of slight offsets from the exact resonance condition. In the extended data in Supplementary fig 4, we present similar data but now include the phase information for clarity. The phase information, representing the instantaneous phase (ϕ) of the spins on the Bloch sphere equator, is particularly revealing and shows the full extent of the intricate micromotion dynamics. This also illustrates the power of our measurement methodology, which can quasi-continuously track phase and amplitude information on the Bloch sphere.

We present two examples: one at a slight off-resonance of δf = 0.02 Hz (Supplementary fig 4A) and another at a more significant offset of δf = 1 Hz (Supplementary fig 4B). Upper panels show $\langle I^x \rangle$, while lower panels show ϕ, with the rails (0, π) referring to the $\hat{\mathbf{x}}, -\hat{\mathbf{x}}$ axes, respectively. The beating observed is exactly at the resonance offset frequency as expected. The phase data particularly reveals multiple strands, corresponding to the micromotion between multiple stroboscopic prethermal plateaus corresponding to each of the interpulse spacings within a single Floquet cycle. The AC field generates a component to the prethermal axis that is transverse to $\hat{x}$ and is rotated by the spin-lock pulses about $\hat{x}$, causing nontrivial micromotion. A more detailed discussion of similar

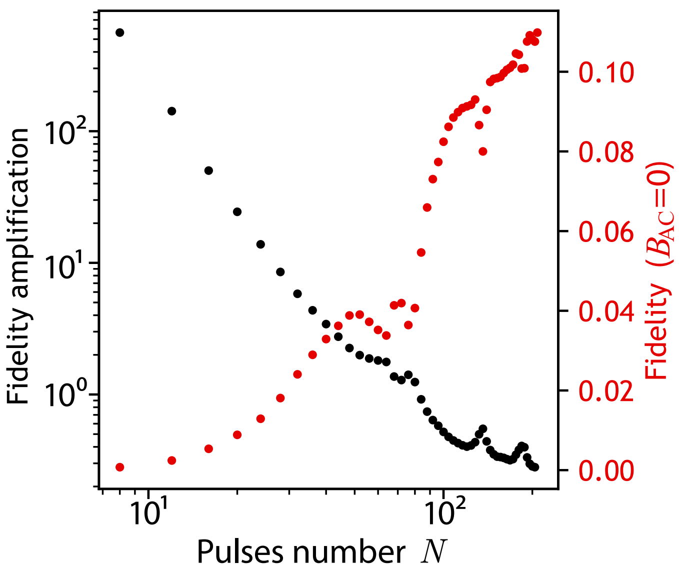

Supplementary fig 3. **Amplification of fidelity** by application of 82.4 μT AC field relative to the zero AC case as a function of number of spin-lock pulses N interspersed between γ_y kicks, shown by black data points corresponding to left axis. For all N, spacings of 36 μs between each pulse are used, and the pulse length for $\gamma_y = \pi$ is 102.5 μs. The AC field increases fidelity by a larger factor for smaller N, while the baseline fidelity without AC (red, right axis) increases with N. For $N > 80$, AC is observed to decrease fidelity, opposite to the coherence extension focused on in this paper.

micromotion can be found in Ref. [36].

C. Sub-harmonic response at large B_{AC}

While the dominant effect of the AC field, shown in Fig. 3A, is the first harmonic response (i.e., a strong extension of lifetime at the resonance frequency f_{res}), we also observe subharmonic responses (dominated by $f_{res}/2$). This occurs when two or more effective γ_y-kick cycles matches one AC field period.

This phenomenon becomes visually apparent at larger B_{AC} values. Supplementary fig 5 studies this response, tracking the frequency for different B_{AC} values. For small B_{AC} values Supplementary fig 5A, the response is dominated by the first harmonic. However, at larger B_{AC} values Supplementary fig 5B-C, subharmonic responses, especially at $f_{res}/2$, become more evident.

D. Sensitivity

As described in Sec. IV, the sensor achieves a sensitivity of 880 pT/$\sqrt{Hz}$ with an applied bias of 415 nT. The sensitivity is worsened with increasing bias as shown in Supplementary fig 6. This is because we find that the shot-to-shot variance in fidelity for DTCs prepared with nominally identical conditions grows with AC amplitude more quickly than the fidelity response to AC perturbations. One possible source of shot-to-shot variance is fluctuations in the initial level of hyperpolarization; it was observed in Ref. [37] that hyperpolarization not only scales the magnetization signal but also affects coherence time. Minimizing such sources of shot-to-shot variance, so that uncertainty in F is instead dominated by the readout noise within in a single shot, could disproportionately improve sensitivity. Not only would this decrease noise σ_F in the fidelity metric at any fixed AC bias, but it would allow operation in a higher bias regime where the response $\partial F/\partial B_{AC}$ to AC perturbation is greater. Mitigation of shot-to-shot variance could be achieved by corrections in offline analysis as well as by improvements to the apparatus (e.g. laser stability). Reducing shot-to-shot variance so that fluctuations σ_F in the fidelity metric at fixed B_{AC} are dominated by the readout of the magnetization would allow us to exploit the stronger response $\partial F/\partial B_{AC}$ at biases around 8 μT. Beyond minimizing shot-to-shot variance, sensitivity could be substantially improved by reducing uncertainty on the fidelity F estimated for a single shot. A simple way to achieve this is increasing sample volume, as the present sample only occupies a small portion of the 8 mm dimensions of the inductive readout coil. Increasing the fill factor of the diamond in the coil could improve sensitivity by a factor of ≈25. The signal-to-noise ratio within the magnetization readout could also be improved by increasing hyperpolarization of the ^{13}C nuclei or enriching their concentration above the 1% natural abundance in the current sample. Noise could also be reduced by upgrades to the readout electronics. With these improvements made in combination, an order-of-magnitude enhancement in sensitivity is expected. The prospective sensitivity is indicated in Supplementary fig 1.

In addition to these technical considerations, we finally note that γ_y pulses play a dual role in determining the sensitivity, as they set both the intrinsic DTC lifetime and the strength of the coupling to the AC field. Because these two effects compete, optimizing the γ_y-pulse duration is nontrivial and remains an important direction for future work.

E. Noise rejection of the DTC sensor

We demonstrate in Fig. 3A that the DTC sensor exhibits a frequency linewidth of 69 mHz. This narrow linewidth allows the sensor to reject off-resonant noise, as shown in Supplementary fig 7. In this experiment, we first apply a bias field of strength $B_{bias} = 3.29\,\mu$T, which is resonant with the γ_y pulses of the DTC sequence. We then introduce AC fields an order of magnitude smaller than the bias field $B_{noise} = 329.6$ nT with varying frequency to illustrate that only the resonant signal affects the sensor significantly, while off-resonant noise has minimal effect.

In Supplementary fig 7, we measure the increase in fidelity when AC fields, with amplitudes an order of magnitude smaller than the bias field and with varying frequencies, are applied along with the bias field. This is compared to the baseline case where only the bias field is present. The plot shows that a marked increase in fidelity only occurs when the AC field is resonant, indicated by the gray dotted line at f_{res}. This result

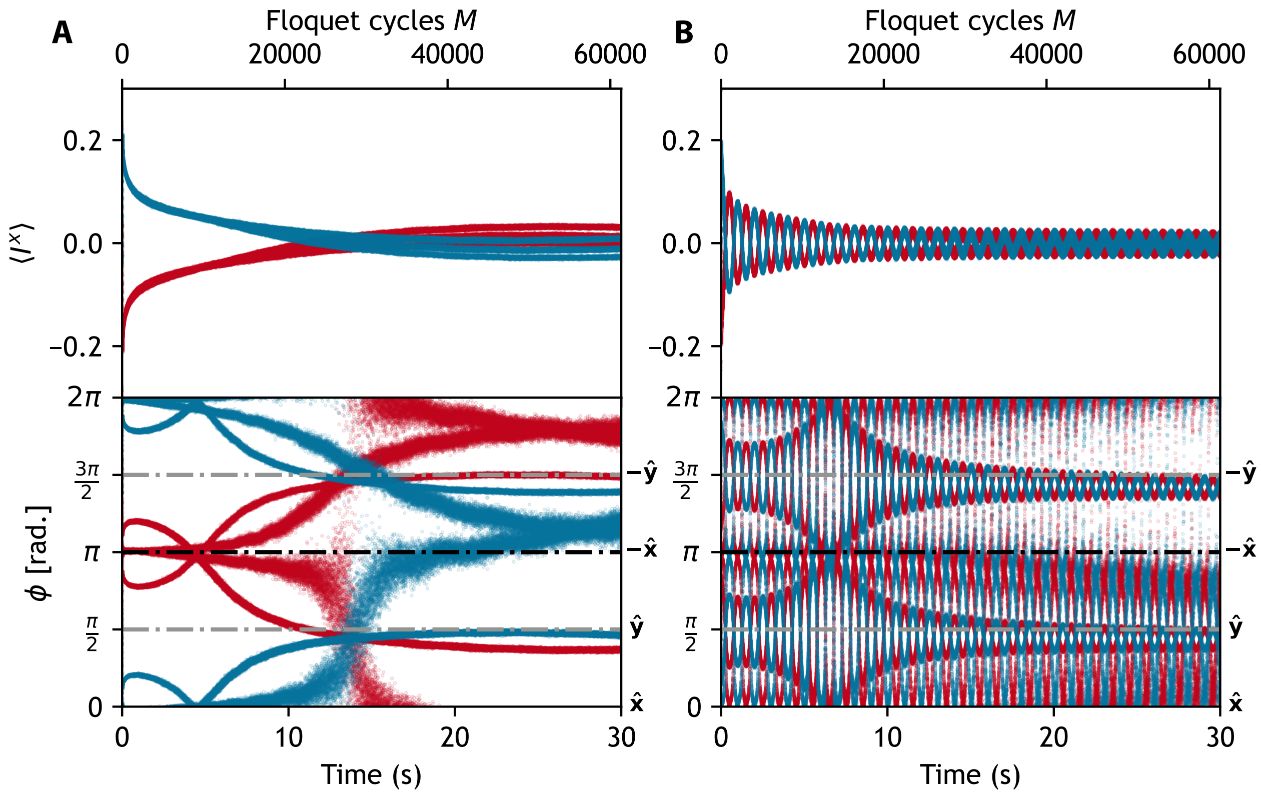

Supplementary fig 4. **Dynamics of DTC with off-resonant AC mapped in phase** ϕ (lower) as well as polarization $\langle I^x \rangle$ (upper), shown for AC frequencies with small offsets of δf=0.02 Hz (A) and δf=1 Hz (B) from resonance. Solid boundaries at ϕ=0, 2π correspond to the $+\hat{\mathbf{x}}$ axis along which spins point upon initiating the DTC, while the dash-dotted black line at ϕ=π indicates the $-\hat{\mathbf{x}}$ axis. Dash-dotted gray lines at ϕ=$\pi/2, 3\pi/2$ indicate the $+\hat{\mathbf{y}}$ and $-\hat{\mathbf{y}}$ axes respectively. Blue points represent measurements after an even number of γ pulses, while red represent measurements after an odd number of flips. Each plot has over 200000 total points, allowing us to rapidly and quasi-continuously track spin motion along the Bloch sphere equator, revealing intricate micromotion dynamics. Data is shown up to t=30 s and 60,000 γ_y pulses (upper axis). Beating at the offset frequency from resonance is observed - after each half beat, the direction of the I^x component is observed to invert. We also observed an increasing magnitude of the I^y component during the sequence. Beyond the separation between even and odd number of γ-kicks, we observe further subdivision into multiple strands associated with micromotion between spacings between each of the $N = 4$ spin lock pulses.

highlights the DTC sensor's capability to detect signals at a specific frequency in a noisy environment containing different frequencies.

F. Estimating magnetic field from the $\hat{\mathbf{z}}$-coil

To generate an AC field aligned with the B_0 =7 T, a secondary coil is positioned inside the NMR probe to generate B-field parallel to $\hat{\mathbf{z}}$. To estimate the B-field applied to the diamond sample, we measure how the peak of the Fourier-transformed free induction decay (FID) signal shifts as the voltage of the Tektronix device is varied. Supplementary fig 8 shows how the shift in peak frequency (f_{peak}), as the B-field from the secondary coil changes with increasing DC voltage from the Tektronix source. The change in frequency, Δf_{peak}, is given by $\Delta f_{\text{peak}} = \gamma_n(1-\sigma)\Delta B = \gamma_n(1-\sigma)(\Delta B/\Delta V)\Delta V$, where γ_n is the gyromagnetic ratio of the ^{13}Cnuclear spin, σ is the chemical shift, ΔV is the change in voltage from the voltage source, and ΔB is the corresponding change in the magnetic field. The slope shown in Supplementary fig 8 corresponds to $\gamma_n(1-\sigma)(\Delta B/\Delta V)$, indicating that a 1V change from the Tektronix source corresponds to approximately 164.85 μT. This is based on the value of $\gamma_n(1-\sigma) = 7$ T/75.38 MHz = 9.29×10^{-8} T/Hz.

G. Three-tone drive

Supplementary fig 9 shows how the normalized $\hat{\mathbf{x}}$-magnetization $\langle I^x \rangle$ changes in time under a three tone drive. The pulse sequence, shown in the inset of Fig. 3C, begins with a $(\frac{\pi}{2})_y$ pulse to tip the ^{13}Cnuclear spins' magnetization to the $\hat{\mathbf{x}}$-axis of their rotating frame. Then, γ_y pulses are applied at two distinct frequencies $2f_{\text{res}}^{(1)}$ and $2f_{\text{res}}^{(2)}$, interleaved with the spin-lock sequence. The signal from ^{13}C nuclear spins is read out between the pulses, while AC fields with a magnitude of B_{AC} = 329.6 nT and varying frequencies are applied.

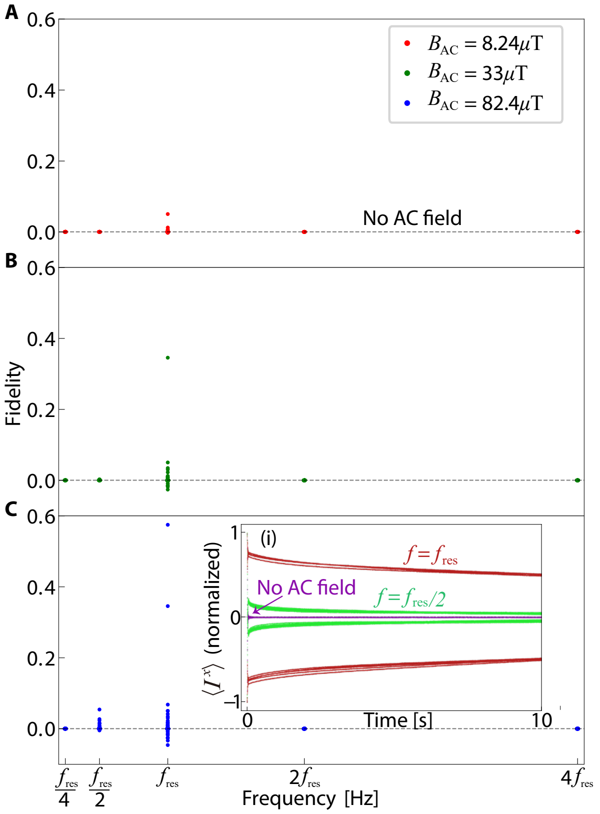

Supplementary fig 5. **Subharmonic response** in the AC field for different AC field amplitudes, following the methodology of Fig. 3A in the main paper, with N=4. (A-B) For smaller B_{AC} values, as indicated in Fig. 3A, the response is dominated by the first harmonic at f_{res}, corresponding to fitting a single AC field period within one Floquet cycle. (C) For larger AC field amplitudes, subharmonic responses appear at $f_{res}/2$, respectively. This corresponds to fitting a single AC field period within two resonance Floquet cycles. (i) *Inset* shows the oscillatory response of $\langle I^x \rangle$ over time for three cases: No Ac field (purple), subharmonic (green), and resonant (red) AC field with a magnitude of $B_{AC} = 82.4\,\mu$T is applied. $(\pi/2)_y$ and θ_x pulses ($\approx 50.73\,\mu$s); $\tau(\approx 36.03\,\mu$s); γ_y pulse ($\approx 101.55\,\mu$s).

We observe resonant responses in two cases: when the AC field frequency is $f^{(1)}_{res}$ and when the anti-nodes of the AC field align with the γ_y pulses at a frequency $2f^{(1)}_{res}$. A similar resonant condition is observed for the AC field at $f^{(2)}_{res}$.

The three-tone drive exhibits a different resonant response compared to the two-tone drive. Notably, regardless of the two resonant conditions, $\langle I^x \rangle$ decreases when an AC field is applied, particularly at later times ($>$ 1.5s). This decline is clearly depicted in the insets of Supplementary fig 9A and Supplementary fig 9B, where more $\langle I^x \rangle$ is preserved in the absence of an AC field compared to the both off-resonant and resonant cases for $f^{(1)}_{res}$ and $f^{(2)}_{res}$. This behavior occurs because, regardless of whether the AC field is applied at $f^{(1)}_{res}$ or $f^{(2)}_{res}$, off-resonant γ_y pulses are always present, preventing the spins from prethermalizing into an effective Hamiltonian.

However, Fig. 3C shows that, experimentally, the signal decreases the least in the two on-resonant cases, compared to when off-resonant AC-fields of the same magnitude are applied. These two distinct resonant responses can be leveraged to realize a two-frequency sensor.

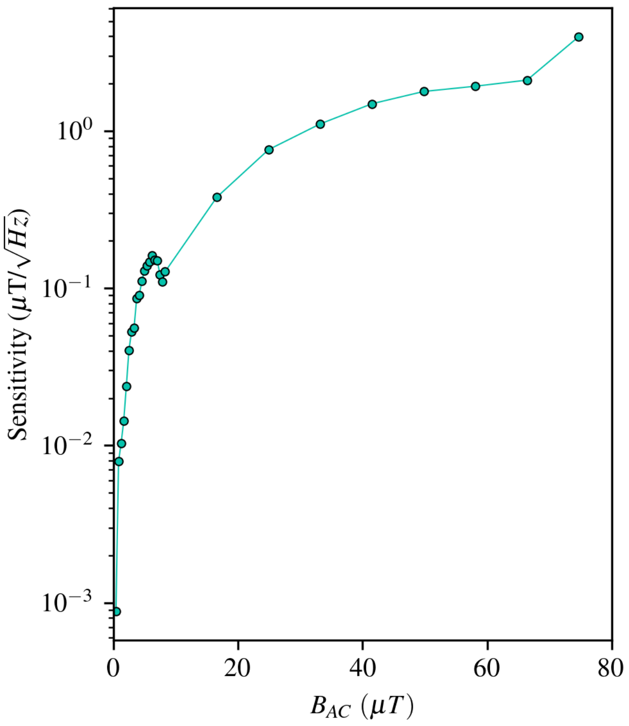

Supplementary fig 6. **Effective AC field sensitivity** measured from fidelity as a function of AC amplitude, following Fig. 2B. Optimum sensitivity is obtained for small AC amplitudes.

H. Spin-lock sensing

Supplementary fig 10 illustrates how the spin-lock sensing data presented in Fig. 3A is obtained. Supplementary fig 10A(i) depicts the sensing protocol introduced in [38]: $(\frac{\pi}{2})_y$ pulse is first applied to tip the ^{13}Cnuclear spins to the x-axis of their rotating frame. A train of θ_x pulse (spin-lock sequence) is applied, and the ^{13}Cnuclear spin signal is read out in between the pulses. Supplementary fig 10A shows how ^{13}Cnuclear spins imprint the applied AC field with a frequency of f = 1000 Hz and a magnitude of $B_{AC} = 8.24\,\mu$T in the phase (ϕ) of their rotating frame. A zoomed-in window in Supplementary fig 10A(ii) clearly displays the imprinted oscillations.

We vary the frequency of the applied AC field and measure the absolute value of the Discrete Fourier Transform (DFT) spectrum, shown in Supplementary fig 10B(i) for f = 1000 Hz. For each frequency, we calculate the mean of the absolute value of the DFT spectrum near its peak, represented as grey points in Supplementary fig 10B. The linear fit is further shown in Supplementary fig 10B as grey dotted line to serve as a visual guide.

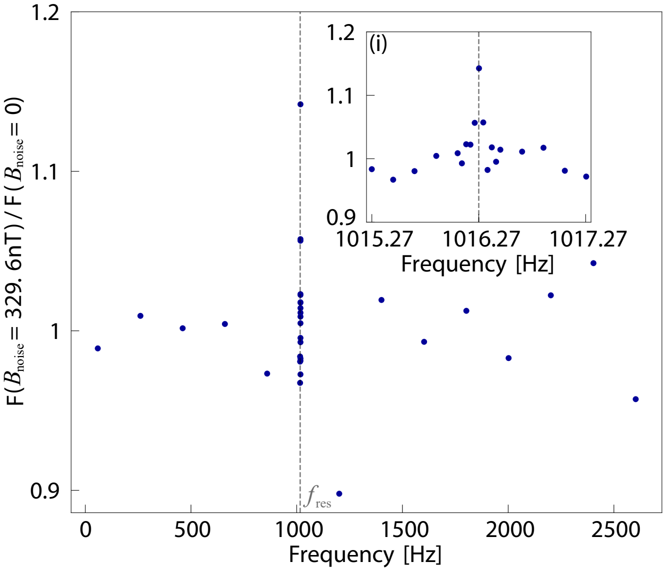

Supplementary fig 7. **Noise rejection of the DTC sensor** The sensor is tuned to a resonant frequency with an applied on-resonant bias AC field of $B_{\text{bias}} = 3.29\,\mu$T, while an order of magnitude smaller noise fields, $B_{\text{noise}} = 329.6$ nT, of varying frequencies are introduced. The plot shows the relative increase in fidelity compared to the baseline fidelity $F(B_{\text{noise}} = 0)$. Off-resonant noise does not significantly affect the baseline, whereas a resonant signal of the same magnitude leads to a noticeable increase in the fidelity. The inset highlights a zoomed-in window of ± 1 Hz around the resonant frequency $f_{\text{res}} =$ 1016.27 Hz. $(\pi/2)_y$ and θ_x pulses ($\approx 51.96\,\mu$s); $\tau(\approx 36\,\mu$s); γ_y pulse ($\approx 104.01\,\mu$s)

The linear profile of the mean of the absolute value of the DFT spectrum near its peak demonstrates that while the spin-lock sensing scheme can detect multiple frequencies with a single pulse parameter, it lacks the frequency selectivity offered by the DTC sensing scheme.

To illustrate the spin-lock sensing scheme's linewidth on resonance as reported in [38], we vary the frequency of the AC field and measure the integrated signal. As shown in Supplementary fig 10C, the linewidth is approximately 340 Hz, which is at least four orders of magnitude larger than the linewidth of the DTC sensing scheme. This indicates that the spin-lock sensing scheme is not limited by the lifetime of the ^{13}C. Note that the reported linewidth in [38] is 224 Hz, acheived using a different set of pulse parameters and an alternative console to generate the $\pi/2$ pulses. The lack of narrow linewidth in the spin-lock sensing highlights the novelty of the DTC sensing scheme, which can be precisely tuned to a desired frequency.

I. Phase-dependence of single-tone DTC

In Supplementary fig 11, we report on the phase dependence of the AC response of the single-tone DTC; see Fig. 4A for details of the sequence. In contrast to the two-tone DTC, see Fig. 2A(ii), for the single-tone DTC, the optimal response is achieved when the AC field and pulse sequence are in-

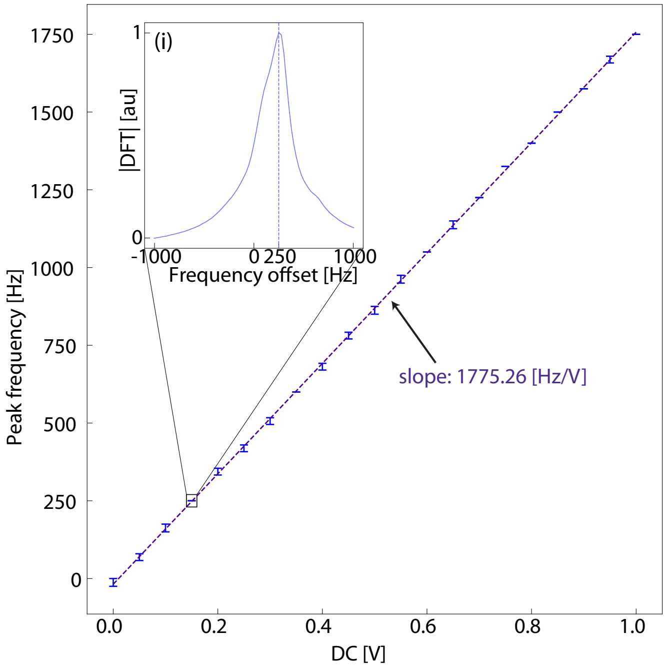

Supplementary fig 8. **Estimating B-field from an AC coil** Free Induction Decay (FID) signals from the coil are measured for different DC voltages applied by the Tektronix source. The FID is mixed down to zero frequency with an on-chip Numerically Controlled Oscillator (NCO). *Inset* (i): Absolute magnitude of the Discrete Fourier Transformed (DFT) of the FID signal for a 0.15 V bias B-field. Each point in the main panel shows the mean of $n = 4$ independent measurements, where each measurement corresponds to the diamond being separately hyperpolarized and acquired in an independent shot; error bars denote mean±SD. A linear fit (purple dotted line) shows the peak frequency shifting linearly with applied DC voltage, with the slope indicating the frequency-shift rate.

phase, $\Phi_{\text{AC}} = 0$. As we detail in Sec. S6 A, this is a direct consequence of the AC field oscillating in the same axis ($\hat{\mathbf{z}}$) as the signal response of the single-tone DTC; therefore, the largest signal is accumulated when no sign change between two $\hat{\mathbf{y}}$-pulses occurs.

S5. NUMERICAL ALGORITHM

We perform (closed-system) quantum simulations of the experimental sequence, detailed in Fig. 1, on a small number L of spins ($L = 15$) using the QuSpin python library[39, 40] and a slightly modified version of the algorithm used in Ref. [9].

System. The experimental setup consists of a macroscopic ensemble of NV-centers each surrounded by a cluster of 1,000-10,000 nuclear spins that are randomly distributed on the vertices of the diamond lattice. The nuclear spins interact via the dipole-dipole coupling

$$H_{\text{dd}} = \sum_{k<\ell}^{L} J_{k\ell} \left(3 I_k^z I_\ell^z - \boldsymbol{I}_k \cdot \boldsymbol{I}_\ell \right) \quad \text{(S 1)}$$

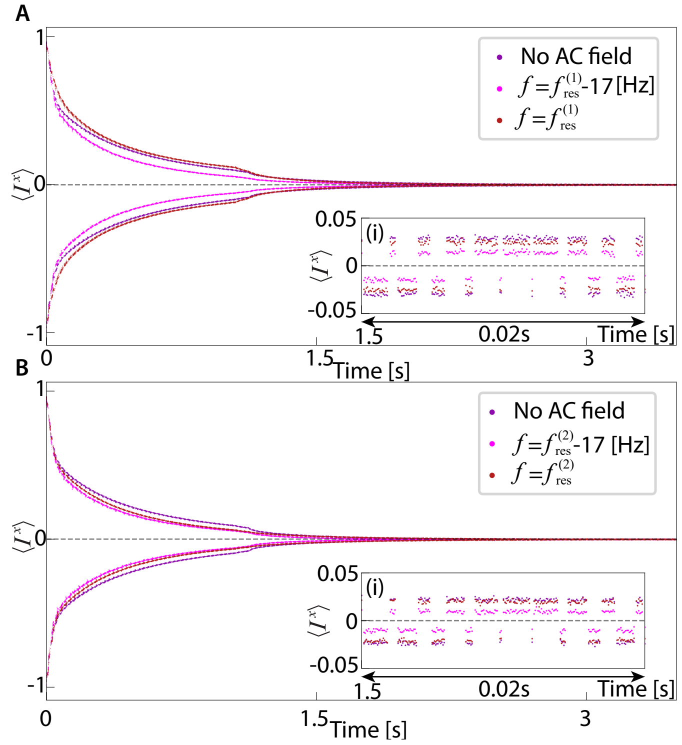

Supplementary fig 9. **Three-tone drive** depicted in the inset of Fig. 3C functions as a two-frequency sensor, detecting two distinct resonant frequencies: $f_{\rm res}^{(1)}$ =208 Hz and $f_{\rm res}^{(2)}$ =250 Hz. (A) Time vs normalized $\hat{\mathbf{x}}$-magnetization, $\langle I^x\rangle$, for the three tone drive under three conditions: no AC field (dark purple), an AC field with frequency $f = f_{\rm res}^{(1)} - 17$ Hz (magenta), and an AC field at frequency $f = f_{\rm res}^{(1)}$ (dark red). (B) Time vs $\langle I^x\rangle$ under similar conditions for $f_{\rm res}^{(2)}$: no AC field(dark purple), an AC field with frequency $f = f_{\rm res}^{(2)} - 17$ Hz (magenta), and AC field at frequency $f = f_{\rm res}^{(2)}$ (dark red). Insets (A)(i) and (B)(i) provide a zoomed-in view of $\langle I^x\rangle$ for the time interval between 1.5 and 1.52 seconds. Both insets show that $\langle I^x\rangle$ is the most stable at later times (> 1.5s) with no-AC field, while off-resonant AC fields cause the most decoherence in the $\hat{\mathbf{x}}$ magnetization.Pulse sequence dimensions listed in Fig. 3C.

with $J_{k\ell} = c_{\rm exp}(3\cos^2\theta_{k\ell} - 1)/r_{k\ell}^3$, where $r_{k\ell}$ is the distance between two spins on sites k and ℓ and $\theta_{k\ell}$ is the angle between the vector connecting the two spins and the direction of the magnetic field ($\hat{\mathbf{z}}$); $c_{\rm exp}$ is a sample-dependent constant.

While the clusters of nuclear spins can be thought of as isolated during the experimental time scale, the effective coherent system size in the experiment exceeds thousands of spins. In stark contrast, our exact numerical simulations are limited to few ($L = 15$) spins due to the exponentially increasing Hilbert space dimension. To mimic the experiment with this small system size we use a specifically tailored random graph instead of placing the spins randomly on a diamond lattice. Concretely, to make the best use of the small system size, we want to avoid (i) spins that are too weakly coupled to the rest of the system and (ii) spins that are too strongly coupled, since both would result in spins being effectively decoupled from the rest of the system. Therefore, we use the procedure from Ref. [9]: Spin

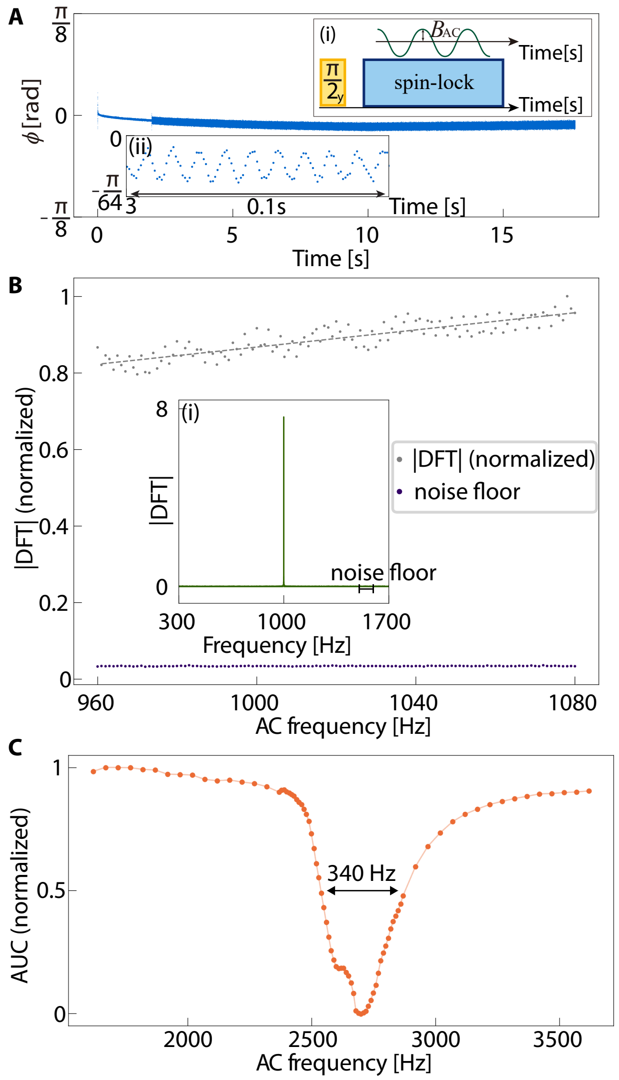

Supplementary fig 10. **Spin-lock sensing** (A) (i) The spin-lock sequence is applied for approximately 17.7 seconds, during which an AC field with $B_{\rm AC} = 8.24\ \mu$T is introduced in the $\pm\hat{\mathbf{z}}$ direction after 2 seconds. The oscillations in the ^{13}Cnuclear spins, induced by the AC field, are imprinted in the phase (ϕ) of their rotating frame. (ii) A zoomed-in view of the data over a 0.1 second window at 3.05 seconds is presented. $(\frac{\pi}{2})_y$ and θ_x pulses ($\approx 52.24\ \mu s$). τ ($\approx 36.03\ \mu s$). (B) Each grey point represents the mean of the Discrete Fourier Transform (DFT) spectrum of the phase signal from ^{13}Cnuclear spins in the vicinity of its peak for different applied AC field frequencies, with the values normalized relative to the maximum across all frequencies. (B)(i) shows the full DFT spectrum for an applied of 1000 Hz, plotted in green. The average signal between 1600 and 1700 Hz, with the frequency range denoted by a black error bar in B(i), is used to measure the noise floor. Dark purple points in (B) indicate the noise floor (normalized using the same maximum value as the grey points) across different AC field frequencies. (C) The FWHM (Full Width at Half-Maximum) corresponds to approximately 340 Hz. Pulse length of the spin lock is $\approx 55.4\ \mu s$, and pulse spacing is $\approx 40.0\ \mu s$.

positions are drawn randomly one by one in a 3D cube such that (a) each spin has a maximal distance $r_{\rm max}$ to at least one other spin and (b) each spin has at least a distance $r_{\rm min}$ to all

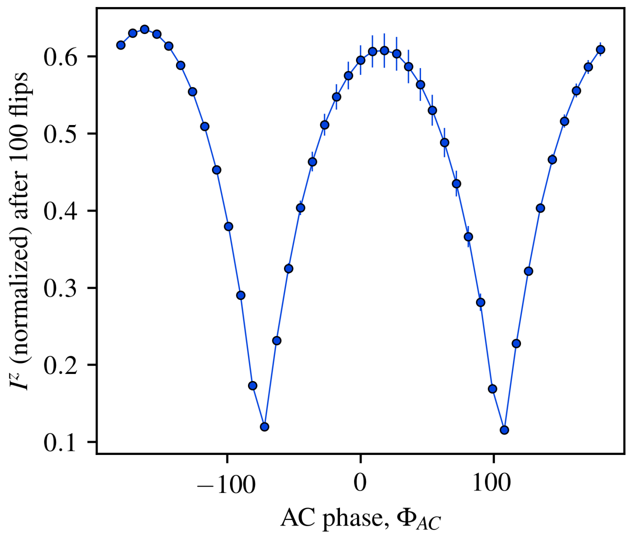

Supplementary fig 11. **Phase dependence of single-tone DTC extension.** In contrast to the two-tone DTC, extension of the single-tone DTC is maximized when AC nodes (rather than extrema) occur during the γ_y pulses. Each data point represents the mean of $n = 4$ independent shots, where each shot corresponds to the diamond being separately hyperpolarized and measured in an independent run; error bars denote mean±SEM. This behavior is explained in Secs. S6 A and S6 B: in the single-tone case the effect arises from the AC field between pulses, whereas in the two-tone case the effective field is generated by the field applied during the pulses.

other spins; thus, avoiding (i) and (ii) respectively. We use $r_{\mathrm{min}} = 0.9$ and $r_{\mathrm{max}} = 1.1$ throughout.

In addition, an important aspect of the 3D dipole-dipole couplings in the experiments performed in this work is the homogeneous distribution of positive and negative couplings, i.e., in a sufficiently large system we find (iii) $\sum_{k<\ell} J_{k\ell} = 0$ as the spins are on-average uniformly distributed on the unit sphere in 3D. This property is in general not fulfilled for the random 3D graph as the number of spins is too small to lead to a uniform distribution. Instead, we enforce this condition by hand using the fact that the sign of the interactions,$(3\cos^2\theta_{k\ell} - 1)$, depends on the orientation with respect to the external z-field direction. Therefore, only after having drawn a random graph we (c) choose the orientation of the entire graph such that (iii) $\sum_{k<\ell} J_{k\ell} = 0$ is fulfilled. To further mitigate finite size effects we average the magnetization dynamics over different realizations of random graphs (usually n_{samples}=50-100 samples).

Finally, in the experiment the initial state after hyperpolarization is $\rho_0 \sim 1 + \mu I^z$ with finite polarization μ. Here, instead we use the fully polarized pure state $|\psi_0\rangle = |\uparrow\dots\uparrow\rangle$ which has been shown to reproduce comparable dynamics at lower computational cost [9].

Unitary Evolution. In the following, we describe the unitary evolution implemented in the numerical simulations. Specifically, the experimental two-tone sequence, see Supplementary fig 12 and main text Fig. 1B, over a single DTC cycle can be recast into the following unitary evolution [36]

$$U_{\mathrm{DTC},\ell} = U_{y,\ell} U_{\mathrm{dd},\ell,N+1} \prod_{k=1}^{N} U_{x,\ell,k} U_{\mathrm{dd},\ell,k}\,, \tag{S 2}$$

with the $\hat{\mathbf{y}}$-pulse

$$U_{y,\ell} = \mathcal{T}\exp\left(-i\int_{(\ell+1)T-\tau_y}^{(\ell+1)T} B_y I^y + B_{\mathrm{AC}}(t) I^z + H_{\mathrm{dd}} \mathrm{d}t\right), \tag{S 3a}$$

and $\hat{\mathbf{x}}$-pulses

$$U_{x,\ell,k} = \mathcal{T}\exp\left(-i\int_{\ell T+k\tau-\tau_x}^{\ell T+k\tau} B_x I^x + B_{\mathrm{AC}}(t) I^z + H_{\mathrm{dd}} \mathrm{d}t\right) \tag{S 3b}$$

and inter-pulse evolution

$$U_{\mathrm{dd},\ell,k} = \exp\left[-i\left(\tau H_{\mathrm{dd}} + \int_{\ell T+(k-1)\tau}^{\ell T+k\tau-\tau_x} \mathrm{d}t B_{\mathrm{AC}}(t) I^z\right)\right], \tag{S 3c}$$

where we used that H_{dd} conserves the z-magnetization, $[I^z, H_{\mathrm{dd}}] = 0$, to solve the time-ordering $\mathcal{T}$ explicitly in the last equation. Note that the AC field varies very slowly in comparison to the length of the $\hat{\mathbf{x}}$ and $\hat{\mathbf{y}}$-pulses, $f_{\mathrm{AC}} \ll 1/\tau_x, 1/\tau_y$. Therefore, we assume a quasi-stationary AC field during those pulses, hence, simplifying the time-ordered integrals to

$$U_{y,\ell} \approx \exp\left[-i\left(\gamma_y I^y + \vartheta^{\mathrm{eff}}_{y;\ell} I^z + H_{\mathrm{dd}}\tau_y\right)\right], \tag{S 4a}$$

with $\vartheta^{\mathrm{eff}}_{y;\ell} = \int_{(\ell+1)T-\tau_y}^{(\ell+1)T} B_{\mathrm{AC}}(t)\mathrm{d}t$,

$$U_{x,\ell,k} \approx \exp\left[-i\left(\theta_x I^y + \vartheta^{\mathrm{eff}}_{x;\ell,k} I^z + H_{\mathrm{dd}}\tau_x\right)\right], \tag{S 4b}$$

with $\vartheta^{\mathrm{eff}}_{x;\ell,k} = \int_{\ell T+k\tau-\tau_x}^{\ell T+k\tau} B_{\mathrm{AC}}(t)\mathrm{d}t$, and

$$U_{\mathrm{dd},\ell,k} \approx \exp\left[-i\left(\vartheta^{\mathrm{eff}}_{\mathrm{dd};\ell,k} I^z + H_{\mathrm{dd}}\tau\right)\right], \tag{S 4c}$$

with $\vartheta^{\mathrm{eff}}_{\mathrm{dd};\ell,k} = \int_{\ell T+(k-1)\tau}^{\ell T+k\tau-\tau_x} \mathrm{d}t B_{\mathrm{AC}}(t) I^z$. The evolution with the approximate unitaries (S 4) can be efficiently implemented using exact diagonalization and is done via the Quspin python package.

Similarly, for the single-tone DTC one can use the same unitary evolution, Eqs. (S 2) and (S 4), by setting τ_x=0. Let us emphasize that, in contrast to previous work [9, 36, 41, 42], we consider the full finite time $\hat{\mathbf{x}}$ and $\hat{\mathbf{y}}$ pulses. While the results in the absence of an AC field are qualitatively independent of the finite time of the pulses, in the presence of the AC field in the two-tone DTC they are vital to account for the observed behavior as described in Sec. S6 B.

S6. FLOQUET ENGINEERING FINITE ENERGY DENSITY

In this section, we will detail the average Hamiltonian analysis for single (S6 A) and two-tone (S6 B) DTC sequences. We

will demonstrate that the lifetime enhancement in both cases is due to the AC field effectively Floquet-engineering a coupling to the DTC order parameter. This coupling introduces a finite energy density for DTC ordered states, thus energetically protecting those states from prethermalization to a featureless, infinite temperature state. In subsection S6 C we summarize the key ingredients required for the AC-induced lifetime extensions of DTC order. Finally, in subsection S6 D we compare our AC scheme to a previously introduced DC scheme [43].

A. One-tone prethermal discrete time crystal

Let us first focus on the conceptually simpler case of single-tone DTC: Here, the analysis is made easier since, the AC field $H_{\text{AC}} = B_{\text{AC}}(t) I^z$ and interactions H_{dd} commute ($[H_{\text{AC}}, H_{\text{dd}}]$=0), such that we can simply integrate the AC field between two consecutive y-pulses. Note that, even in the absence of the AC field for perfect y-pulses (γ_y=π) the symmetry-protected DTC is in principle infinitely long-lived, due to the perfect conservation of I^z. However, for finite ($\gamma_y \neq \pi$), but small ($\gamma_y \approx \pi$) deviations $\epsilon = \gamma_y - \pi$, this conservation law is broken leading to a fast decay of the polarization with heating rate Γ_e determined through Fermi's Golden rule as

$$\Gamma_e \propto (\epsilon/T)^2 \tag{S 5}$$

Such deviations are ubiquitous in the experiment due to spatially varying magnetic fields within the macroscopic sample [41, 42].

We will now describe how an AC field can stabilize the PDTC order against symmetry-breaking terms, and, in fact, lead to an exponential enhancement in lifetime. Integrating the AC field in between two y-pulses the dynamics is described by

$$U = \left(U_y U_{+z} U_{\text{dd}}\right) \left(U_y U_{-z} U_{\text{dd}}\right) \left(U_y U_{+z} U_{\text{dd}}\right) \ldots, \tag{S 6}$$

where $U_y = \exp\left(-i\gamma_y I^y\right)$, $U_{\pm z} = \exp\left(\pm i\overline{B}\tau I^z\right)$ with magnitude of integrated AC field $\overline{B} \propto B_{\text{AC}}$ and interactions $U_{\text{dd}} = \exp(-i\tau H_{\text{dd}})$. For simplicity, in Eq. (S 6), we have neglected the AC field during the $\hat{\mathbf{y}}$ pulses, corresponding to the limit of infinitely fast pulses. A direct comparison with the numerical simulations shows a qualitatively good agreement and justifies this approximation, see Supplementary fig 13A. However, neglecting the finite duration of the pulses is not generally valid as we will see in the two-tone driving case.

Note that, in the special case $\gamma_y = \pi$, we have, $U_y U_{\text{dd}} = U_{\text{dd}} U_y$ and $U_y U_{\pm z} = U_{\mp z} U_y$ such that after $2N$ y-pulses the dynamics are given by $U(2N) = (U_{+z} U_{\text{dd}})^{2N}$. For imperfect y-pulses, $\epsilon \neq 0$, the dynamics after even numbers of y-pulses is effectively described by $H_{\text{eff}}^{1,\text{AC}} = H_{\text{dd}} + \overline{B} I^z + B_y I^y$. While $H_{\text{eff}}^{1,\text{AC}}$ no longer preserves the z-polarization it admits a finite energy density for the initial state $\mathcal{E} = \left\langle H_{\text{eff}}^{1,\text{AC}} \right\rangle_{\rho_0} / L = \overline{B}\mu$. Therefore, the system prethermalizes to a finite temperature state $\rho_{\mathcal{T}} \propto e^{-H_{\text{eff}}^{1,\text{AC}}}/\mathcal{T}$, with $\mathcal{T}$ such that $\text{Tr}\left(H_{\text{eff}}^{1,\text{AC}} \rho_{\mathcal{T}}\right) = \mathcal{E}$.

While Floquet heating leads to a slow increase of the effective temperature ($\mathcal{T}$) [44, 45] this process is exponentially suppressed in the driving period T,

$$\Gamma_e^{\text{AC}} \propto \exp(-1/JT)\,, \tag{S 7}$$

for (quasi-)short-range interacting systems [46–48]; this includes the sign-changing dipole-dipole interactions despite the interactions falling off as $1/r^3$ [49]. This is in stark contrast to the polynomial suppression due the Fermi's Golden Rule heating (S 5) in the absence of the AC field; thus, introducing the AC field leads to an exponential increase in the scaling of the lifetime with decreasing period T. The AC induced lifetime enhancement is also supported by numerical simulations, taking into account the full dynamics, see Supplementary fig 13A.

B. Two-tone prethermal discrete time crystal

We will now turn to the two-tone DTC. The two-tone DTC without an AC field is described in detail in Ref. [9]. Let us only summarize the key aspects here.

In the absence of an AC field, the two-tone DTC drive leads to the unitary evolution per DTC-cycle (period T)

$$U_{\text{2DTC}} = U_y U_{\text{dd}} \prod_{\ell=0}^{N-1} U_x U_{\text{dd}}\,, \tag{S 8}$$

with $U_y = \exp\left[-i\left(\gamma_y I^y + \tau_y H_{\text{dd}}\right)\right]$ and $U_x = \exp\left[-i\left(\theta_x I^x + \tau_x H_{\text{dd}}\right)\right]$ and $U_{\text{dd}} = \exp(-i\tau H_{\text{dd}})$; note that, the product, $\prod_{\ell=0}^{N-1} O_\ell$, is time-ordered running from right to left, $\prod_{\ell=0}^{N-1} O_\ell = O_{N-1} O_{N-2} \ldots O_1 O_0$ Note that both θ_x and γ_y are of order $O(1) = O(\tau^0, T^0)$, thus, preventing the application of the Baker-Campbell-Hausdorff (BCH) formula. Instead, to take care of the strong $\hat{\mathbf{x}}$-pulses we consider a toggling frame expansion, i.e., using $U_x^{-1} U_x = 1$ we can rewrite the spin-locking dynamics (taking away the $\hat{\mathbf{y}}$ pulse) of Eq. (S 8) as

$$\begin{aligned} U_y^\dagger U_{\text{2DTC}} &= U_{\text{dd}} \prod_{\ell=0}^{N-1} U_x U_{\text{dd}} \\ &= U_{\text{dd}} U_x^N \left(\prod_{\ell=0}^{N-1} U_x^{-\ell} U_{\text{dd}} U_x^\ell \right) \\ &= U_x^{N+1} \left(\prod_{\ell=0}^{N} U_x^{-\ell} U_{\text{dd}} U_x^\ell \right) \\ &= U_x^{N+1} \left[\prod_{\ell=0}^{N} \exp\left(-i\tau \tilde{H}_{\text{dd},\ell}\right) \right], \end{aligned} \tag{S 9}$$

where we introduced $\tilde{H}_{\text{dd},\ell} = U_x^{-\ell} H_{\text{dd}} U_x^\ell$ in the last line. Since, $\left\|\tilde{H}_{\text{dd},\ell}\right\| = \|H_{\text{dd}}\|$, with respect to the Frobenius norm, all instantaneous generators in the product in the final expression in Eq. (S 9) are of order $\|H_{\text{dd}}\|\tau$ allowing for application of the BCH formula,

$$\prod_{\ell=0}^{N} \exp\left(-i\tau \tilde{H}_{\text{dd},\ell}\right) \approx \exp\left(-i\tau \sum_{\ell=0}^{N} \tilde{H}_{\text{dd},\ell}\right)$$

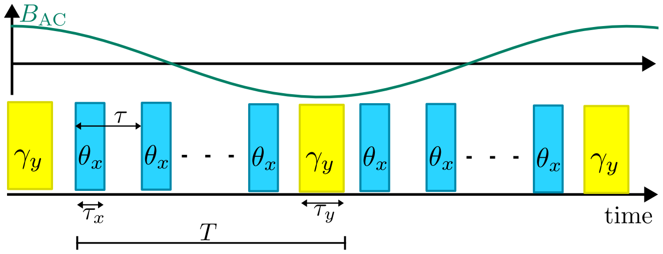

Supplementary fig 12. **Detailed DTC sequence.** Sketch of DTC sequence with individual pulse duration τ_x and τ_y, as well as, spin-locking period τ and full period T; extended version of Fig 1B(i).

up to $O(\tau)$ corrections [9]. One can show that if $N+1=4n$ ($n \in \mathbb{Z}$, and $\theta_x = \pi/2$ exactly, that the lowest order Floquet Hamiltonian

$$H_{\mathrm{SL}} \equiv \frac{1}{N}\sum_{\ell=0}^{N} \tilde{H}_{\mathrm{dd},\ell} = \frac{1}{2}\sum_{k<\ell} J_{k\ell}\left(3I_k^x I_\ell^x - \boldsymbol{I}_k \cdot \boldsymbol{I}_\ell\right) \quad \text{(S 10)}$$

corresponds to the spin-lock Hamiltonian H_{SL} that preserves the I^x magnetization, $[H_{\mathrm{SL}}, I^x] = 0$.

For all other values of N one can show that the emergent $U(1)$-symmetry is broken already in the lowest order $O(T^0)$. However, the explicit symmetry-breaking term is suppressed in N as $O(1/N)$, such that symmetry-breaking effects are still suppressed out to longer times. Therefore, the spin-locking sequence leads to an emergent $U(1)$-symmetry and the dynamics are well-described by replacing the spin-locking sequence $U_{\mathrm{dd}} \prod_{\ell=0}^{N-1} U_x U_{\mathrm{dd}}$ of duration $T_{\mathrm{spin-lock}} = (N+1)\tau - \tau_x$ by $U_{\mathrm{spin-lock}} = e^{-iT_{\mathrm{spin-lock}} H_{\mathrm{SL}}}$. Then, the role of the $\hat{\mathbf{y}}$-pulses is the same as in the single-tone DTC case in Sec. II C.

The key difference to the single-tone DTC, however, is the fact that the AC-field and DTC-oscillation axes are orthogonal. Thus, the coupling to the DTC order parameter is not immediately evident. In fact, naively one would expect the AC field to cause either an effective z-field that enhances symmetry breaking or be averaged out by the DTC sequence. Indeed, in the limit of instantaneous y-pulses this is exactly the case, as demonstrated below.

However, in the experiment a significant portion of the period is spent applying the $\hat{\mathbf{y}}$-pulses—the duration τ_y of the $\hat{\mathbf{y}}$-pulses exceeds the evolution time τ between pulses, $\tau_y > 2\tau$—such that the AC-field also impacts these pulses. Note that, the period of the AC field is much larger than the duration of the $\hat{\mathbf{y}}$-pulses. Therefore, we will assume that the AC field is quasi-constant during the $\hat{\mathbf{y}}$-pulses, such that the dynamics during the pulse are effectively described by

$$U_y(n;t) = e^{-it\left(\gamma_y I^y + (-1)^n \alpha I^z\right)/\tau_y}, \quad \text{(S 11)}$$

where n indicates the n'th applied $\hat{\mathbf{y}}$-pulse, τ_y is the length of the y-pulse, $0 < t < \tau_y$ and $\alpha = \left|\int B_{\mathrm{AC}}(t)\mathrm{d}t\right|$ is the accumulated angle in z. For simplicity, we have disregarded the action of the interactions during the $\hat{\mathbf{y}}$ pulses which lead to minor corrections in the final result (see simulations below).

Going to a rotating frame with respect to the strong I^y field and rotating back the dynamics are exactly described by

$$U_y(n;t) = e^{-i\gamma_y I^y}\,\mathscr{T} e^{-i(-1)^n \alpha \int_0^{\tau_y} \frac{\mathrm{d}t}{\tau_y}\left[\cos(\gamma_y t/\tau_y) I^z - \sin(\gamma_y t/\tau_y) I^x\right]}, \quad \text{(S 12)}$$

where $\mathscr{T}$ refers to time-ordering. To leading order, $O(\frac{\alpha}{\gamma_y})$, we can approximate Eq. (S 12) by

$$U_y(n;t) \approx e^{-i\gamma_y I^y} e^{i\frac{(-1)^n \alpha}{\gamma_y} I^x}, \quad \text{(S 13)}$$

where $\alpha \propto B_{\mathrm{AC}}\tau_y$. Hence, the AC z-field on top of the finite time y-pulse leads to an effective AC-x field. Therefore, the two-tone DTC is formally similar to the single-tone DTC. Indeed, in Supplementary fig 13B we also provide numerical evidence for the AC-induced lifetime enhancement, taking into account the full dynamics.

In contrast, to the single-tone DTC where the $U(1)$-symmetry, $[I^z, H_{\mathrm{dd}}] = 0$, is exact to all orders, for the two-tone DTC the $U(1)$-symmetry is only quasi-conserved, i.e., higher order terms $O(JT)$ break the symmetry. Thus, the heating without AC field is expected to follow a power law in the period $\Gamma_e \propto (JT)^2$. In contrast, via engineering a finite energy density via the AC field, this decay follows the usual exponentially suppressed Floquet heating decay rate $\Gamma_e^{\mathrm{AC}} \propto \exp(-1/JT)$. This is the origin of the increase in lifetime, produced by the AC field, and observed in the experiment.

Limit of instantaneous pulses. To emphasize the importance of the finite duration pulses, we demonstrate that in the case of instantaneous pulses, the AC-field exactly cancels out at the resonance condition. Specifically, let us consider the fine-tuned case $\theta_x = \pi/2$, $\gamma_y = \pi$, with ideal angle $\Phi_{\mathrm{AC}} = \pi/2$ and on-resonance condition $f_{\mathrm{AC}} = f_{\mathrm{res}}$. Moreover, let us take a closer look into the DTC evolution in the presence of an AC

field over two cycles:

$$
\begin{aligned}
U^2_{\mathrm{2DTC}} &= U_y U_{\mathrm{dd}} U_{z,2N+2} \left(\prod_{\ell=1}^{N} U_x U_{\mathrm{dd}} U_{z,N+1+\ell} \right) \\
&\qquad \cdot U_y U_{\mathrm{dd}} U_{z,N+1} \left(\prod_{\ell=1}^{N} U_x U_{\mathrm{dd}} U_{z,\ell} \right) \\
&= U_y \left(\prod_{\ell=0}^{N} U_x^{\ell} U_{\mathrm{dd}} U_{z,N+2+\ell} U_x^{-\ell} \right) \\
&\qquad \cdot U_y \left(\prod_{\ell=0}^{N} U_x^{\ell} U_{\mathrm{dd}} U_{z,\ell+1} U_x^{-\ell} \right) \\
&= U_y \left(\prod_{\ell=0}^{N} \tilde{U}_{\mathrm{dd},\ell} \tilde{U}_{z,N+2+\ell} \right) U_y \left(\prod_{\ell=0}^{N} \tilde{U}_{\mathrm{dd},\ell} \tilde{U}_{z,\ell+1} \right) \\
&\overset{O(T^0)}{\approx} U_y \left(\prod_{\ell=0}^{N} \tilde{U}_{\mathrm{dd},\ell} \right) \left(\prod_{\ell=0}^{N} \tilde{U}_{z,N+2+\ell} \right) \\
&\qquad \cdot U_y \left(\prod_{\ell=0}^{N} \tilde{U}_{\mathrm{dd},\ell} \right) \left(\prod_{\ell=0}^{N} \tilde{U}_{z,\ell+1} \right) \\
&\overset{(\mathrm{S}\,10)}{\approx} U_y e^{-iT_{\mathrm{spin-lock}} H_{\mathrm{SL}}} \left(\prod_{\ell=0}^{N} \tilde{U}_{z,N+2+\ell} \right) \\
&\qquad \cdot U_y e^{-iT_{\mathrm{spin-lock}} H_{\mathrm{SL}}} \left(\prod_{\ell=0}^{N} \tilde{U}_{z,\ell+1} \right) \\
&\overset{O(T^0)}{\approx} e^{-i2T_{\mathrm{spin-lock}} H_{\mathrm{SL}}} U_y \left(\prod_{\ell=0}^{N} \tilde{U}_{z,N+2+\ell} \right) U_y \left(\prod_{\ell=0}^{N} \tilde{U}_{z,\ell+1} \right)
\end{aligned}
$$

where the definitions of $U_{z,\ell}$ are given below, and in the last line we used that $\left[U_y, H_{\mathrm{SL}}\right] = 0$ for $\gamma_y = \pi$.

Hence, in summary, to lowest order $O(T^0)$ the interaction and single-particle fields decouple

$$
U^2_{\mathrm{2DTC}} \approx e^{-i2T_{\mathrm{spin-lock}}} U^2_{\mathrm{sp}} \,, \qquad (\mathrm{S}\,14)
$$

with single-particle unitary

$$
U^2_{\mathrm{sp}} = U_y U_{z,2N+2} \left(\prod_{\ell=1}^{N} U_x U_{z,N+1+\ell} \right) U_y U_{z,N+1} \left(\prod_{\ell=1}^{N} U_x U_{z,\ell} \right) \,, \qquad (\mathrm{S}\,15)
$$

where $U_y = \exp(-i\gamma_y I^y)$, $U_x = \exp(-i\theta_x I^x)$, and $U_{z,\ell} = \exp(-iB_\ell I^z)$ with integrated field $B_\ell = \int_{(\ell-1)\tau}^{\ell\tau} B_{\mathrm{AC}}(t)\mathrm{d}t$. Therefore, in the following we can focus on the single-particle contributions only, introducing the interactions only in the end.

Note that, (i) by symmetry of the cosine function we have $B_{N+1+\ell} = B_{N+1-\ell}$ and (ii) for $\gamma_y = \pi$ we have $U_y f(I^x, I^z) U_y = U_y^2 f(-I^x, -I^z)$ for any function f, i.e., in particular $U_y U_{x,z} U_y = U_y^2 U^\dagger_{x,z}$. Thus, we can rewrite Eq. (S 15) as

$$
\begin{aligned}
U^2_{\mathrm{sp}} &= U_y^2 U^\dagger_{z,1} \left(\prod_{\ell=1}^{N} U_x^\dagger U^\dagger_{z,N+1-\ell} \right) U_{z,N+1} \left(\prod_{\ell=1}^{N} U_x U_{z,\ell} \right) \\
&= U_y^2 U^\dagger_{z,1} \left(U_x^\dagger U^\dagger_{z,2} \ldots U_x^\dagger U^\dagger_{z,N} U_x^\dagger \underline{U^\dagger_{z,N+1}} \right) \underline{U_{z,N+1}} \\
&\qquad \cdot \left(U_x U_{z,N} \ldots U_x U_{z,2} U_x U_{z,1} \right) \\
&= U_y^2 U^\dagger_{z,1} U_x^\dagger U^\dagger_{z,2} \ldots U_x^\dagger U^\dagger_{z,N} \underline{U_x^\dagger U_x} U_{z,N} \ldots U_x U_{z,2} U_x U_{z,1} \\
&= \ldots \\
&= U_y^2 = -1 \,,
\end{aligned}
$$

where we repeatedly apply the unitarity of U_x and $U_{z,\ell}$, i.e., $U_x^\dagger U_x = 1 = U^\dagger_{z,\ell} U_{z,\ell}$. Therefore, by Eq. (S 14), the full two-cycle DTC evolution is given by

$$
U^2_{\mathrm{2DTC}} \approx -e^{-i2T_{\mathrm{spin-lock}}} \,, \qquad (\mathrm{S}\,16)
$$

to lowest order $O(T^0)$, which is equivalent to the two-cycle unitary in the absence of an AC field $B_{\mathrm{AC}} = 0$.

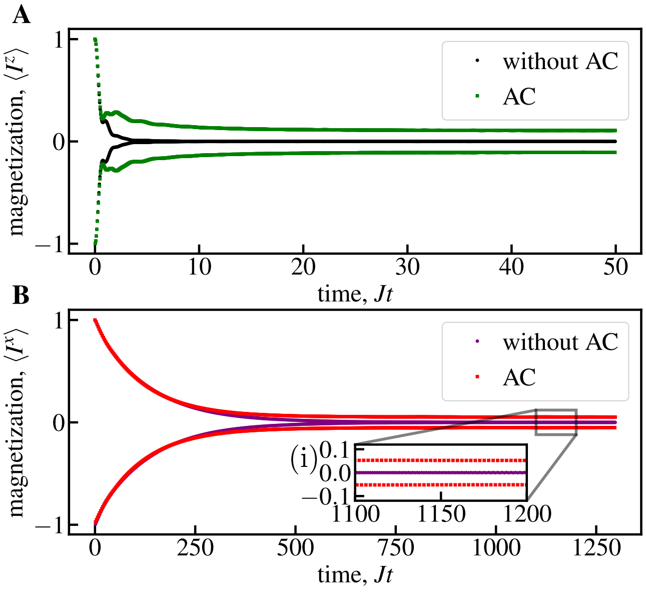

Supplementary fig 13. **Numerical simulation of AC induced lifetime enhancement** for single-tone (**A**) and two-tone (**B**) DTC, with $\Phi_{\mathrm{AC}} = 0, \pi/2$, respectively, using the algorithm detailed in Sec. S5. We use $N = 16$, $\gamma_y = 0.98\,\pi$, $\theta_x = \pi/2$, $J\tau = 0.025$, $\tau_y{=}3\tau{=}2\tau_x$ and $B_{\mathrm{AC}} = J/\pi$. **A** Magnetization $\langle I^z \rangle$ dynamics without (black) and with (green) AC field. **B** Magnetization $\langle I^x \rangle$ dynamics without (purple) and with (red) AC field; (i) zoom into late time regime. For both DTCs the additional AC field leads to an increase in signal and lifetime, in perfect agreement with experiments, see Figs. 1 and Fig. 4.

C. Summary of AC-induced signal enhancement

While the analysis above focused on dipolar interacting spins in a 3D system driven by a specific Floquet-sequence, the

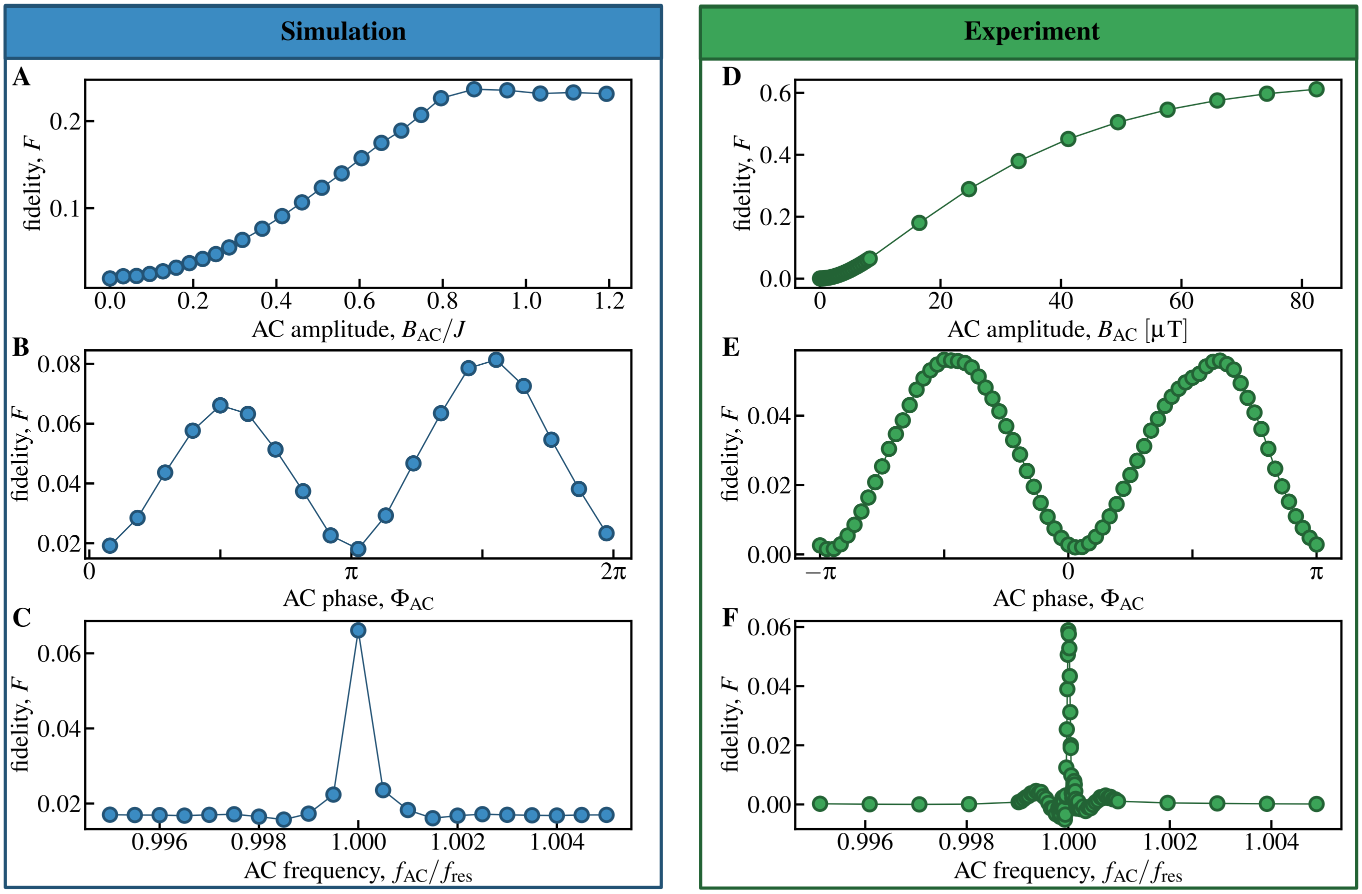

Supplementary fig 14. **Numerical simulation of properties of AC induced signal enhancement** for two-tone DTC. We depict the dependence of the fidelity metric on the properties of AC drive: (**A**), amplitude B_{AC} sweep. The fidelity metric increases monotonically with the AC amplitude B_{AC}. (**B**), phase Φ_{AC} sweep, Φ_{AC} measured as shown in Fig. 2A. data shows a roughly $\sin^2$-like dependence on the phase with maximal signal achieved around $\Phi_{AC} = \pi/2, 3\pi/2$. (**C**), frequency f_{AC} sweep. The signal enhancement is a strongly resonant effect around the intrinsic DTC frequency f_{res}. (**D**-**F**), are the experimental analogs for (**A**-**C**); experimental data is same as shown in main text Fig. 2 and Fig. 3. The simulated results are in qualitative agreement with the experimental results. Static parameters for simulation are as in Supplementary fig 13, and experimental details are found in main text Fig. 2 and Fig. 3.

results apply more broadly. For example, the key role of interactions is to drive thermalization in agreement with the eigenstate thermalization hypothesis (ETH); however, this may also be achieved by considering a thermodynamic ensemble or by using open systems.

In a nutshell, the key ingredients are: (i) (emergent) symmetry-protected period doubling response; (ii) the system should (pre-)thermalize in agreement with ETH, (iii) high-temperature initial state such that lifetime is limited by symmetry-breaking terms; and (iv) ability to (effectively) couple the system to the DTC order parameter, e.g., via Floquet engineering. Note that, since the DTC order parameter oscillates in time the coupling-inducing term will generally be time-dependent as well. Then, by adding this time-varying coupling one can exponentially extend the lifetime of the PDTC order using the procedure presented above.

Let us further point out that the exponential suppression of Floquet heating in the drive frequency $1/T$ only applies to short-range and effectively short-range interacting systems [46, 47]. Note that in general the critical exponent for effective short-range interactions is $\alpha = d$, where the interactions scale as $J \propto 1/r^{\alpha}$ and d is the spatial dimension. Hence, our dipole-dipole interacting system is critical long-range interacting and not short-range interacting. However, it was shown that for sign-changing interactions the critical exponent is indeed $\alpha = d/2$ [49]. Thus, the sign-changing dipole-dipole interacting system behaves as a short-range system for the purpose of Floquet heating, i.e., displaying exponential suppression of Floquet heating in the drive frequency.

Finally, let us emphasise that in order to observe the scaling with period T one has to (i) reduce the pulse-durations τ_y and τ_x accordingly, while (ii) not only keeping the accumulated angles γ_y and θ_x fixed but also decrease their deviations from the ideal values $(\gamma_y - \pi)$, $\theta_x \propto T$. The required accuracy and high-power during the pulses makes observing the exponential scaling in the current experimental setup technically challenging.

D. AC vs DC field comparison

Let us emphasize that the idea of using finite energy density to stabilize $U(1)$-DTC order is not new to this work. In Ref. [43], the authors proposed to stabilize a sequence similar to the single-tone DTC above by adding a DC field, $B_{\mathrm{DC}} = hI^z$ with strength h, in the time-window where no $\hat{\mathbf{y}}$ pulses are applied. An advantage of the DC field is that, depending on the strength of the field one can, (i) engineer a finite energy density ($h\tau \approx \pi/2$) similar to the procedure in this work or (ii) restore the broken $U(1)$-symmetry ($h\tau = \pi$) by averaging out errors in γ_y. However, away from $h\tau = n\pi/2$ ($n \in \mathbb{Z}$) the $\gamma_y \approx \pi$ pulses 'echo-out' the DC field, thus, requiring the persistent application of strong magnetic fields which can be experimentally challenging, due to the high-power required and the potential heating of the sample.

In contrast, by applying an AC field the 'echoing-out' is avoided, such that lifetime enhancement occurs for any finite value of the AC amplitude. This not only provides a practical advantage but forms the basis of the AC sensing application. Moreover, the AC sensing scheme generalizes to the two-tone DTC; a similar extension for the DC protocol is not immediately clear, since, the additional $\theta_x = \pi/2$ spin-locking pulses would 'echo-out' the $\hat{\mathbf{z}}$-DC field and persistently applying an $\hat{\mathbf{x}}$-DC field would interfere with the signal readout.

S7. CHARACTERISTICS OF AC ENHANCED SIGNAL

From the theoretical derivation in Sec. S6 B we concluded that the AC field leads to an additional I^x-magnetization in the effective Hamiltonian $H_{\mathrm{eff}} = H_{\mathrm{SL}} + B_{\mathrm{eff}} I^x$ which causes prethermalization to a finite energy and magnetization state that is protected during the prethermal plateau. To lowest order, the effective field is given by integrating the AC field during the y-pulse, $B_{\mathrm{eff}} = \int_{t_0}^{t_0+\tau_y} B_{\mathrm{AC}}(t)\mathrm{d}t/\gamma_y$. As we show below, this is sufficient to qualitatively explain the observed response of the DTC to different properties of the AC field $B_{\mathrm{AC}}(t) = B_{\mathrm{AC}} \sin(2\pi f_{\mathrm{AC}} t + \Phi_{\mathrm{AC}})$, i.e., the phase Φ_{AC}, amplitude B_{AC} and frequency f_{AC} of the AC drive. We support our theoretical findings with numerical simulations, see Supplementary fig 14A-C.

To this end, let us explicitly derive the magnetization in the prethermal plateau. Assuming a high-temperature state, $J\mathcal{T} \gg 1$, we can write the prethermal state as $\rho_{\mathcal{T}} \approx 1 - H_{\mathrm{eff}}/\mathcal{T}$. Then, the inverse temperature can be determined by quasi-energy conservation as $\langle H_{\mathrm{eff}}\rangle_{\rho_0}/L = \mu B_{\mathrm{eff}} \overset{!}{=} \langle H_{\mathrm{eff}}\rangle_{\rho_{\mathcal{T}}} = -\left[B_{\mathrm{eff}}^2 + \|H_{\mathrm{SL}}\|^2\right]/\mathcal{T}$, hence,

$$\mathcal{T}^{-1} = -\frac{\mu B_{\mathrm{eff}}}{B_{\mathrm{eff}}^2 + J_{\mathrm{spin-lock}}^2}\,, \tag{S 17}$$

where μ is the magnetization of the initial state and where we defined $J_{\mathrm{spin-lock}}^2 = \|H_{\mathrm{SL}}\|^2$ which measures the effective interaction strength of the spin-locking Hamiltonian. Consequently, the magnetization per spin in the prethermal plateau is given by

$$\langle I^x\rangle_{\rho_{\mathcal{T}}}/L \approx -\mathcal{T}^{-1} B_{\mathrm{eff}} = \mu \frac{B_{\mathrm{eff}}^2}{B_{\mathrm{eff}}^2 + J_{\mathrm{spin-lock}}^2}\,. \tag{S 18}$$

Note that, the Floquet heating dynamics after prethermalization correspond to an exponential decay of the magnetization magnitude in the prethermal plateau to zero, $|\langle I^x(t = nT)\rangle| \sim e^{-\Gamma_e^{\mathrm{AC}} t}\left|\langle I^x\rangle_{\rho_{\mathcal{T}}}\right|$. This allows for a simple relationship between the fidelity metric and magnetization in the prethermal plateau in the resonant case: there, the fidelity metric corresponds to time-integrating the absolute value of the signal, i.e., $F \propto \sum_{n=0}^{N}(-1)^n \langle I^x(nT)\rangle = \sum_n |\langle I^x(nT)\rangle| \sim \langle I^x\rangle_{\rho_{\mathcal{T}}} \sum_n e^{-\Gamma_e^{\mathrm{AC}} nT}$. Thus, the fidelity metric is directly proportional to the magnetization in the prethermal plateau, i.e., $F = c\,\langle I^x\rangle_{\rho_{\mathcal{T}}} + F_0$ with some proportionality constant c and a shift F_0 originating from the pre-thermalization dynamics.

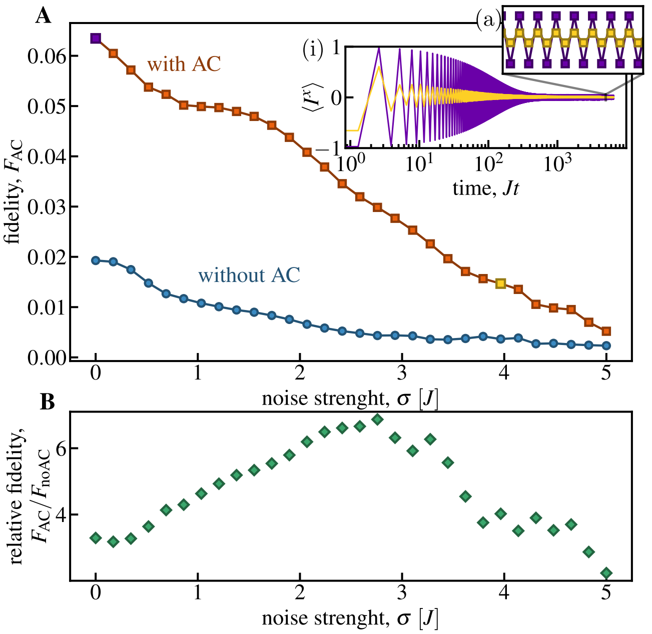

Supplementary fig 15. **Numerical simulation of noise resilience of AC induced signal enhancement** for two-tone DTC. **A**, fidelity metric F for increasing on-site noise strength σ for DTC without (blue circles) and with (orange squares) AC field, see Sec. S7 D for definition of noise. The noise strength reaches up to five times the strength of the median coupling. (i), DTC dynamics for specific points indicated in A, representing no noise (purple) and large noise (yellow), $\sigma \approx 4\,J$. (a), zoom into late-time dynamics; even at large noise levels the period doubling dynamics remain observable, however, with decreased amplitude. **B**, Relative increase in fidelity comparing the case of additional and no AC field. The prethermal DTC order with and without AC field, is resilient to strong levels of on-site disorder; i.e., period doubling dynamics persist although with reduced amplitude. Notably, for moderate noise levels, the AC enhanced DTC is less affected by disorder resulting in an enhanced relative signal with increasing disorder. Other parameters are as in Supplementary fig 13.

A. Amplitude

Note that, the effective field is directly proportional to the AC field amplitude $B_{\rm eff} \propto B_{\rm AC}$. Further, notice that for small AC, and hence small effective fields $B_{\rm eff}$ ($B_{\rm eff} \ll J_{\rm spin-lock}$), the magnetization (S 18) increases quadratically with the field strength $\langle I^x \rangle_{\rho_{\mathcal{T}}} \propto B_{\rm eff}^2$. This is in excellent agreement with the experimental (Supplementary fig 14D) and numerical results (Supplementary fig 14A).

With increasing field strength the ($B_{\rm eff} \geq J_{\rm spin-lock}$) the magnetization increase stalls and converges to a finite value, namely its initial value $\langle I^x \rangle_{\rho_{\mathcal{T}}} \overset{B_{\rm eff}\to\infty}{\longrightarrow} \mu L$. This trend is in agreement with the observed experimental and theoretical results. However, note that the slowing down observed in the numerical simulation, see Supplementary fig 14A, is stronger than the experimentally observed, see Supplementary fig 14D, and analytically predicted form; this is likely a finite system artefact caused by the field becoming comparable to the spectral width of the system.

B. Phase

Notice that if the AC field averages to zero during the $\hat{\mathbf{y}}$-pulses the effective field vanishes $B_{\rm eff} = 0$; thus, the dynamics agree with those in the absence of the AC field. Further, assuming a quasi-constant AC field the effective field is given by $|B_{\rm eff}| = |\sin(\Phi_{\rm AC})| B_{\rm AC} \tau_y / \gamma_y$ which takes its largest value around $\Phi_{\rm AC} = \pm\pi/2$ and vanishes around $\Phi_{\rm AC} = 0, \pi$ in agreement with the strongest and weakest signal observed in the experiment, Fig. 2A. Moreover, the functional dependence of the magnetization in the weak field regime, $\langle I^x \rangle_{\rho_{\mathcal{T}}} \propto B_{\rm eff}^2 \propto \sin^2(\Phi_{\rm AC})$, matches well in lowest order with experimental (Supplementary fig 14E), and numerical result (Supplementary fig 14B).

C. Frequency

The dependence on the frequency $f_{\rm AC}$ can be understood as follows. For simplicity, we focus on the regime $\Phi_{\rm AC} = \pi/2$. In the far off-resonant regime the accumulated AC field during the $\hat{\mathbf{y}}$-pulses oscillates wildly, thus averaging to zero over a few cycles. In the near resonance regime, i.e., when the difference in frequency is small $\delta f = |f_{\rm AC} - f_{\rm res}| \ll f_{\rm AC}$, we may assume a separation of time-scales $B_{\rm AC}(t) = \cos(2\pi f_{\rm AC} t) = \cos(2\pi f_{\rm res} t)\cos(2\pi \delta f t) + \sin(2\pi f_{\rm res} t)\sin(2\pi \delta f t)$. As we have seen above, the sine contribution can be neglected as it integrates to zero during the $\hat{\mathbf{y}}$-pulses. Thus, the field is given by $B_{\rm AC}(t) \sim \cos(2\pi \delta f t)\cos(2\pi f_{\rm res} t)$, and hence the effective field attains a slowly varying component $B_{\rm eff}(t) = \cos(2\pi \delta f t) B_{\rm eff}$. If the field varies slowly enough, the magnetization follows the change in external field adiabatically without changing the effective temperature, $\langle I^x \rangle_{\rho_{\mathcal{T}}}(t) \approx -\mathcal{T}^{-1} B_{\rm eff}(t)$, thus, leading to the observed beating, in agreement with the experiment Supplementary fig 14F and simulations Supplementary fig 14C. As discussed in the main text, the narrow linewidth is thus a result of time-integrating the signal over the lifetime T_2'.

D. Noise resilience

We use our numerical simulations to explore the robustness of the AC enriched PDTC towards errors in the pulse sequence and local fields on the spins. This is a common source of noise in experimental setups, due to spatial inhomogenities in the magnetic field and imperfections in tuning the $\hat{\mathbf{y}}$ and $\hat{\mathbf{x}}$ pulses. Both the PDTC order and thermalization are expected to be resilient towards these errors, leading to enhanced sensing capabilities as imperfections in the sensor do not reduce sensitivity. While the lack of local control in the experimental apparatus prevents a detailed exploration of this robustness, we can use our numerical simulations to investigate resilience of the sensing protocol.

Specifically, we consider constant-in-time but spatially-varying errors on the $\hat{\mathbf{x}}$ and $\hat{\mathbf{y}}$ pulses, as well as additional on-site fields in $\hat{\mathbf{z}}$-direction, i.e., we replace $\theta_x I^x \to \sum_\ell (\theta_x + \tau_x \chi_\ell) I_\ell^x$, $\gamma_y I^y \to \sum_\ell (\gamma_y + \tau_y \eta_\ell) I_\ell^y$ and $H_{\rm dd} \to H_{\rm dd} + \sum_\ell \zeta_\ell I_\ell^z$ where χ_ℓ, η_ℓ, ζ_ℓ are uniformly distributed numbers in $[-\sigma/2, +\sigma/2]$.

We find a resilience of the response up to noise strengths exceeding the dipolar couplings strength, i.e., $\sigma > J$, see Supplementary fig 15. In fact, for moderate noise strength ($\sigma \leq 3\,J$), the AC enriched DTC seems more robust than the DTC without an AC field, thus leading to relative increase in fidelity metric with increasing noise. However, the overall decrease in signal will result in a smaller T_2' and, thus, less narrow linewidth. In summary, the numerical simulations suggest that moderate levels of disorder in the system have marginal effects on the observed PDTC order.

E. Dependence on spin density

A key characteristic of the ^{13}C-nuclear spin sensor is the abundance of ^{13}C, i.e., the density of nuclear spins, n. While we use a diamond with natural abundance (≈1 %) of ^{13}C in all our experiments, further enrichment is technically possible, providing another parameter to tune the properties of our sensing scheme. Let us emphasize that estimating the impact of the nuclear spin density on the DTC sensing scheme is challenging due to the interplay of multiple factors, specifically:

1. increasing the density of nuclear spins by a factor κ, $n \mapsto \kappa n$ while considering the same sample size increases the net signal by the same factor, $S \mapsto \kappa S$;

2. at the same time, the interspin interaction increases by the same factor, $J \mapsto \kappa J$, which reduces the decoherence time, $T_2 \mapsto T_2/\kappa$, and will also affect the Floquet heating rate. While the precise scaling dependence of the heating rate on the relative frequency, ω/J, is not known, previous experiments are consistent with a power law $T_2' \propto (\omega/J)^2$ [9, 50], hence, $T_2' \mapsto T_2'/\kappa^2$, if the lifetime is limited by Floquet heating;

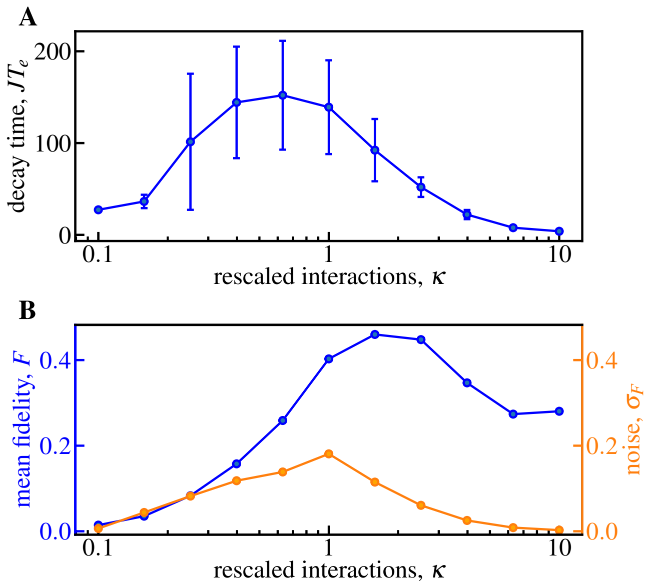

Supplementary fig 16. **Simulated scaling of lifetime and fidelity with spin density without AC field.** We simulate the quantum dynamics as described in Sec. S5 for $L = 15$ spins for various rescalings of the median coupling $J \mapsto \kappa J$, corresponding to a change in spin-density $n \mapsto \kappa n$. **A**, $1/e$-lifetime, T_e, for different interaction strengths show non-monotonous behavior, peaking at finite $\kappa \neq 0, \infty$ (here around $\kappa \approx 0.6$). Each data point represents the mean of 10 independent disorder-graph simulations, and error bars denote the standard deviation of lifetime over different seeds, estimated from the standard deviation of the signal S via $\sigma_{T_e} = \dot{S}\sigma_S \mid_{t=T_e}$. **B**, fidelity metric F (blue) and its standard deviation σ_F (orange) computed using Eq. (1) in the main text. Each fidelity point is likewise the mean of 10 disorder-graph simulations, with error bars indicating the corresponding standard deviation. Fixed-volume estimates were obtained via $F = \kappa F_{L=15}$ and $\sigma_F = \sqrt{\kappa}\sigma_{F,\,L=15}$. Analogously to the decay time, the mean fidelity and its standard deviation show a non-monotonous behavior, peaking at a finite value of $\kappa \neq 0, \infty$; notably, all three quantities peak at different values of κ. We choose $N = 16$, $\gamma = 0.98\pi$; remaining parameters match Supplementary fig 13.

3. conversely, if the lifetime is limited by noise, the relative noise strength σ/J decreases, $\sigma/J \mapsto \kappa^{-1}\sigma/J$, extending the lifetime and increasing the fidelity, see also Sec. S7 D;

4. additionally, the relative AC amplitude also decreases, $B_{AC}/J \mapsto \kappa^{-1}B_{AC}/J$, naively reducing the response of the DTC sensor to the same change in AC amplitude.

Moreover, the spin density may impact the hyperpolarization mechanism and decoherence induced via coupling to $P1$ and NV^--centers; however, precisely how increasing the spin density will affect either mechanism is unclear, since both are difficult to describe quantitatively from first principles. Regardless, we expect the sensitivity to be minimal in both extremes of non-interacting and infinitely strongly interacting nuclear spins; in the former case, there is no interaction-induced stability of the DTC response away from $\gamma_y = \pi$, and in the latter case strong Floquet heating will quickly melt the

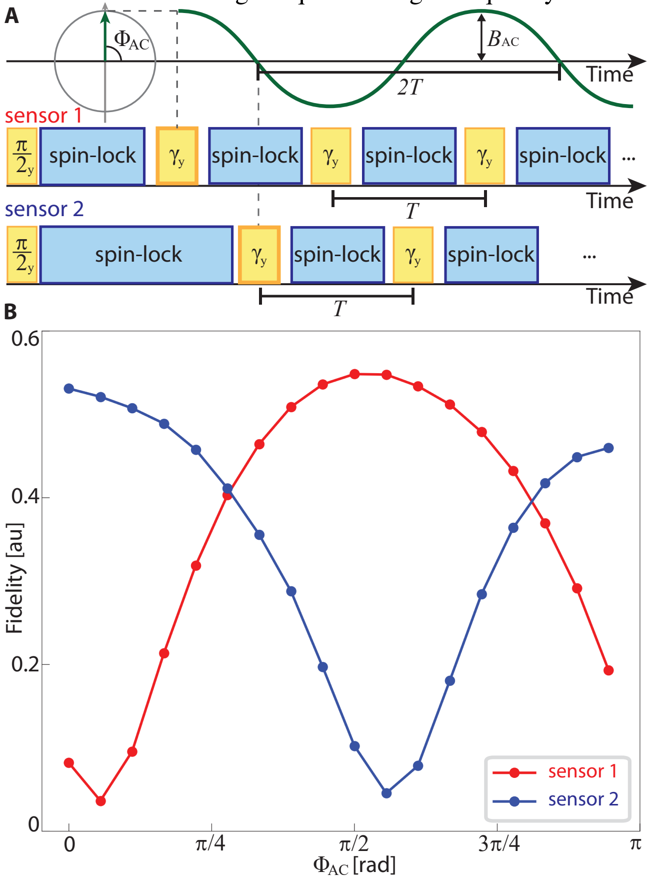

Supplementary fig 17. **Schematic for detecting field with unknown phase using two (or multiple) sensors.**, **A,** Two-sensor sensing schematic. An AC field of known frequency ($(2T)^{-1}$), but unknown phase (Φ_{AC}) and amplitude (B_{AC}) is sensed by two sensors (sensor 1 and sensor 2). The two sensors have γ_y pulses are offseted by $T/2$ to leverage the fidelity's characteristic to uniquely determine B_{AC}. **B,** Two sensors' response depending on the phase of the AC-field (Φ_{AC}). Sensor 1 and sensor 2 are initialized such that the γ_y pulses are offset by $T/2$. The response of sensor 1 (shown in red) and sensor 2 (shown in blue), depends on the phase of the AC-field (Φ_{AC}). Here, $B_{AC} = 41.2\,\mu T$, $T \approx 517.6\,\mu s$, γ_y pulses ($\approx 112.5\,\mu s$), θ_x pulses ($\approx 56.2\,\mu s$), and pulse separation τ ($\approx 36.0\,\mu s$).

DTC order. However, away from these extreme cases the sensitivity increases and peaks at some finite interaction strength $J^\star$; the precise value of this sweet spot will depend on the precise details of the sensor.

Lemma: Consider an AC-field with unknown amplitude $B^{(1)}$ and phase $\Phi^{(1)}$ is applied to the DTC sensor. We show that: *the fidelity values $F(B_{AC} = B^{(1)}, \Phi_{AC} = \Phi^{(1)})$ and $F(B_{AC} = B^{(1)}, \Phi_{AC} = \Phi^{(1)} + \pi/2)$ uniquely determine the amplitude $B^{(1)}$ and the phase $\Phi^{(1)}$ (up to [0, $\pi/2$]) of the AC field.*

Let $B^{(1)}$ and $\Phi^{(1)}$ be the amplitude and the phase of the unknown AC magnetic field.
The fidelity of the discrete time crystal with an AC magnetic field is denoted as $F(B_{\mathrm{AC}}, \Phi_{\mathrm{AC}})$, where B_{AC} is the amplitude and Φ_{AC} is the phase of the field.
$F(B_{\mathrm{AC}}, \Phi_{\mathrm{AC}})$ has two properties.

[A] $\forall \Phi_{\mathrm{AC}} \in [0, \pi]$, $F(B_{\mathrm{AC}}, \Phi_{\mathrm{AC}})$ increases monotonically as B_{AC} increase;

[B] $\forall B_{\mathrm{AC}}$, $F(B_{\mathrm{AC}}, \Phi_{\mathrm{AC}})$ monotonically increases as Φ_{AC} increases for $\Phi_{\mathrm{AC}} \in [0, \pi/2]$ and monotonically decreases as Φ_{AC} increases for $\Phi_{\mathrm{AC}} \in [\pi/2, \pi]$.

Properties [A] and [B] hold because the pulse overlap between the AC magnetic field and the γ_y pulses increases as B_{AC} increases, and the pulse overlap increases as Φ_{AC} increases for $\Phi_{\mathrm{AC}} \in [0, \pi/2]$ and decreases for $\Phi_{\mathrm{AC}} \in [\pi/2, \pi]$. We ignore the slight phase shift in the experimental data, but it can easily be incorporated into the proof by adjusting the bounds of Φ_{AC}.
We assume the response is symmetric between $\Phi_{\mathrm{AC}} \in [0, \pi]$ and $\Phi_{\mathrm{AC}} \in [\pi, 2\pi]$, as shown in Fig. 2A(ii) for $\gamma_y = 0.98$. If not, four sensors would be required instead of two to uniquely determine the phase.
Proof:
We now show by proof of contradiction that $B^{(1)}$ and $\Phi^{(1)}$ (up to $[0, \pi/2]$) are uniquely determined if one measures $F(B^{(1)}, \Phi^{(1)})$ and $F(B^{(1)}, \Phi^{(1)} + \pi/2)$.
Let us assume that there exist $B^{(2)}$ and $\Phi^{(2)}$ such that

$$F(B^{(1)}, \Phi^{(1)}) = F(B^{(2)}, \Phi^{(2)}) \tag{L 1}$$

and

$$F(B^{(1)}, \Phi^{(1)} + \pi/2) = F(B^{(2)}, \Phi^{(2)} + \pi/2)\,, \tag{L 2}$$

where $B^{(1)} \neq B^{(2)}$ or $\Phi^{(1)} \neq \Phi^{(2)}$; this would correspond to observing the same measurement for the two $\pi/2$-shifted sensor for different measurement signals.
Without loss of generality, we assume $0 \leq \Phi^{(1)}, \Phi^{(2)} \leq \pi/2$.

Further, let us focus on the case $B^{(1)}_{\mathrm{AC}} < B^{(2)}_{\mathrm{AC}}$; the case $B^{(1)}_{\mathrm{AC}} > B^{(2)}_{\mathrm{AC}}$ follows from similar arguments. Since F is monotonically increasing for both B_{AC} [A] and Φ [B] for $\Phi \leq \pi/2$, the case $B^{(1)}_{\mathrm{AC}} < B^{(2)}_{\mathrm{AC}}$ and Eq. (L 1) necessitate that $\Phi^{(1)} > \Phi^{(2)}$. Hence, also $\Phi^{(1)} + \pi/2 > \Phi^{(2)} + \pi/2$, such that the monotonic decrease of F for $\Phi \geq \pi/2$ implies that

$$F(B^{(1)}_{\mathrm{AC}}, \Phi^{(1)} + \pi/2) < F(B^{(1)}_{\mathrm{AC}}, \Phi^{(2)} + \pi/2)\,, \tag{L 3}$$

note that, we consider the same B_{AC}-value. Likewise, since we consider $B^{(1)}_{\mathrm{AC}} < B^{(2)}_{\mathrm{AC}}$ and F monotonically increasing in B_{AC} we find

$$F(B^{(1)}_{\mathrm{AC}}, \Phi^{(2)} + \pi/2) < F(B^{(2)}_{\mathrm{AC}}, \Phi^{(2)} + \pi/2)\,, \tag{L 4}$$

where the phase $\Phi = \Phi^{(2)} + \pi/2$ is fixed. Combining Eqs. (L 3) and (L 4), we find

$$F(B^{(1)}_{\mathrm{AC}}, \Phi^{(1)} + \pi/2) < F(B^{(2)}_{\mathrm{AC}}, \Phi^{(2)} + \pi/2)\,,$$

which contradicts the assumption (L 2). Hence, the conditions [A] and [B], and measuring $F(B_{\mathrm{AC}}, \Phi)$ and $F(B_{\mathrm{AC}}, \Phi + \pi/2)$, uniquely determine both the amplitude B_{AC} and the phase Φ of the AC field, up to [0, $\pi/2$] the phase.
□

Therefore, we have shown that using two $\pi/2$-shifted sensor is sufficient to uniquely determine the magnitude of an AC signal without requiring the knowledge of its phase.

Numerical Analysis of the spin density. Since a thorough experimental analysis of the role of nuclear spin density on the sensing capabilities is technically challenging, instead, we focus on a numerical analysis. Specifically, as our numerical

analysis is constrained to small system sizes, we consider a fixed number of spins ($L = 15$) and study the dependence of key characteristics of the experiment on the median coupling strength $J \mapsto \kappa J$, with scaling parameter κ. This corresponds to changing the density and volume of the system simultaneously, $n \mapsto \kappa J$ and $V \mapsto V/\kappa$. To obtain the scaling for fixed volume we consider the extrapolation of the signal to $S \mapsto \kappa S$. To rescale the standard deviation of the signal, we note that the main source of noise in the simulation is the fluctuation over different samples of random graphs; therefore, we expect a self-averaging effect and hence a scaling of the standard deviation according to $\sigma_S \mapsto \sqrt{\kappa}\sigma_S$. This is a major difference compared to the experimental apparatus, where we believe the noise to be dominated by the readout circuit; hence, in the experiment, scaling the signal S scales the signal-to-noise ratio (SNR) by the same factor. Therefore, we generally expect better sensitivity scaling compared to the simulations below, at least up to a certain threshold where the SNR becomes limited by shot noise.

In Supplementary fig 16A and B, we report on the simulated dependence of the DTC lifetime and fidelity metric, respectively, as a function of the interaction rescaling κ in the absence of any AC field; note that, $\kappa = 1$ corresponds to the case studied in the previous sections. In good agreement with the quantitative analysis above, we find that the lifetime decreases drastically for very weak ($\kappa \ll 1$) and very strong interactions ($\kappa \gg 1$), peaking at some intermediate value (here $\kappa \approx 0.6$) which strongly depends on the details of the sequence, sich as the noise strength, the pulses angle γ and the spin-locking angle θ_x, as well as the duration of the pulses. We observe a similar behavior for the fidelity measure and the noise of the fidelity measure. However, the maxima of these different quantities do not coincide. As all three quantities, lifetime, signal, and noise, combined, determine the functionality of the sensor, finding the optimal operation point with respect to spin density is a complex task and will depend on the experimental details of the sensor.

S8. SENSING AC FIELD WITH AN UNKNOWN PHASE

We have demonstrated the measurement of the amplitude of the AC field and the corresponding sensitivity of the DTC sensor when the phase of the AC field (Φ_{AC}) is fixed at $\pi/2$. However, determining B_{AC} for an AC field with an unknown phase presents a challenge, as the fidelity of the DTC sensor is influenced by both the amplitude and the phase of the AC field.

Here, we demonstrate how utilizing two (or more) sensors can accurately measure and determine the amplitude B_{AC} of an AC field, even when its phase Φ_{AC} is unknown. Supplementary fig 17A illustrates the dual-sensor setup. By offsetting the γ_y pulses by $T/2$ between the two sensors, where T is the period of the DTC sequence, we can obtain two fidelity measurements: $F(B_{AC} = B^{(1)}, \Phi_{AC} = \Phi^{(1)})$ and $F(B_{AC} = B^{(1)}, \Phi_{AC} = \Phi^{(1)}_{AC} + \pi/2)$. Here, $F(B_{AC}, \Phi_{AC})$ represents the measured fidelity given the amplitude B_{AC} and the phase Φ_{AC} of the applied AC field.

Supplementary fig 17B shows the response of the two sensors to an AC field with amplitude B_{AC} across different phases Φ_{AC}. By leveraging two key properties of the fidelity – its monotonic increase with AC field amplitude B_{AC} for all phases Φ_{AC}, and its peak occurring at $\Phi_{AC} = \pi/2$ (disregarding a minor experimental phase offset) – offsetting the two sensors by $T/2$ facilitates the unique determination of B_{AC}.

The following lemma demonstrates that we can uniquely determine both the magnitude $B^{(1)}$, and the phase $\Phi^{(1)}$ of the AC field using the fidelity values $F(B_{AC} = B^{(1)}, \Phi_{AC} = \Phi^{(1)})$ and $F(B_{AC} = B^{(1)}, \Phi_{AC} = \Phi^{(1)} + \pi/2)$.

For practical usage, the response of the DTC sensor needs to be characterized for different values of Φ_{AC} and B_{AC}. This involves measuring the fidelity of the DTC across a range of phases and field strengths to approximate the function $F(B_{AC}, \Phi_{AC})$. If the sensor is operated at a bias AC field, the fidelity of the DTC at that bias AC field must be characterized for different B_{AC} and Φ_{AC}.

Given that the wavelength of the AC fields of interest is at least thousands of meters, the additional phase offset due to spatial separation of the sensors is negligible. For the detection of persistent signals, a single sensor could be initialized twice with two different phases.

The sensor's performance diminishes when not operated at the optimal phase, $\Phi_{AC} = \pi/2$. In a two-sensor scheme, sensitivity is expected to increase by approximately $\sqrt{2}$ in the worse case scenario, where the AC field has a phase of $\Phi_{AC} = \pi/4$ for sensor 1 and aliased to $\pi/4$ for sensor 2, due to fidelity measurement aliasing that restricts unique phase determination to $[0, \pi/2]$. We predict a sensitivity increase roughly by a factor of $\sqrt{2}$, because the overlap between γ_y pulses and the AC field scales as $1/\sqrt{2}$ when the phase of the AC field is $\Phi_{AC} = \pi/4$, compared to the optimal phase $\Phi_{AC} = \pi/2$. Using more than two sensors can further enhance sensitivity in the worst-case scenario.

From the lemma, it can be inferred that the phase Φ_{AC} (up to $[0, \pi/2]$) of an AC field can be detected using this two-sensor scheme, since Φ_{AC} is also uniquely determined with B_{AC}. Characterizing $\partial F/\partial \Phi_{AC}$ for various B_{AC} to determine sensor sensitivity to phase is an interesting problem for future investigation.

In addition to the two-sensor scheme, an off-resonance sensing approach can potentially be used to mitigate the sensor's precise phase dependence. Extended coherence is observed for DTCs with a slightly off-resonant AC field applied, but with an additional beating effect (shown in Fig 3A(ii)). This beating signal could be leveraged to detect fields that are slightly off the DTC frequency, where the phase dependence would no longer be significant.

Furthermore, we can exploit the fact that maximum response is achieved when there is a maximum overlap between the AC field and the γ_y pulses. We can initiate the DTC sequence slightly off-resonant to the AC field and monitor for maximum beating (when $\mathrm{d}\langle I_x \rangle/\mathrm{d}t = 0$). At this point, we adjust the sequence to be on-resonance, calibrating the phase at $\Phi_{AC} = \pi/2$, and proceed with the normal DTC sensing scheme. We leave the characterization of off-resonance sensing for future work.

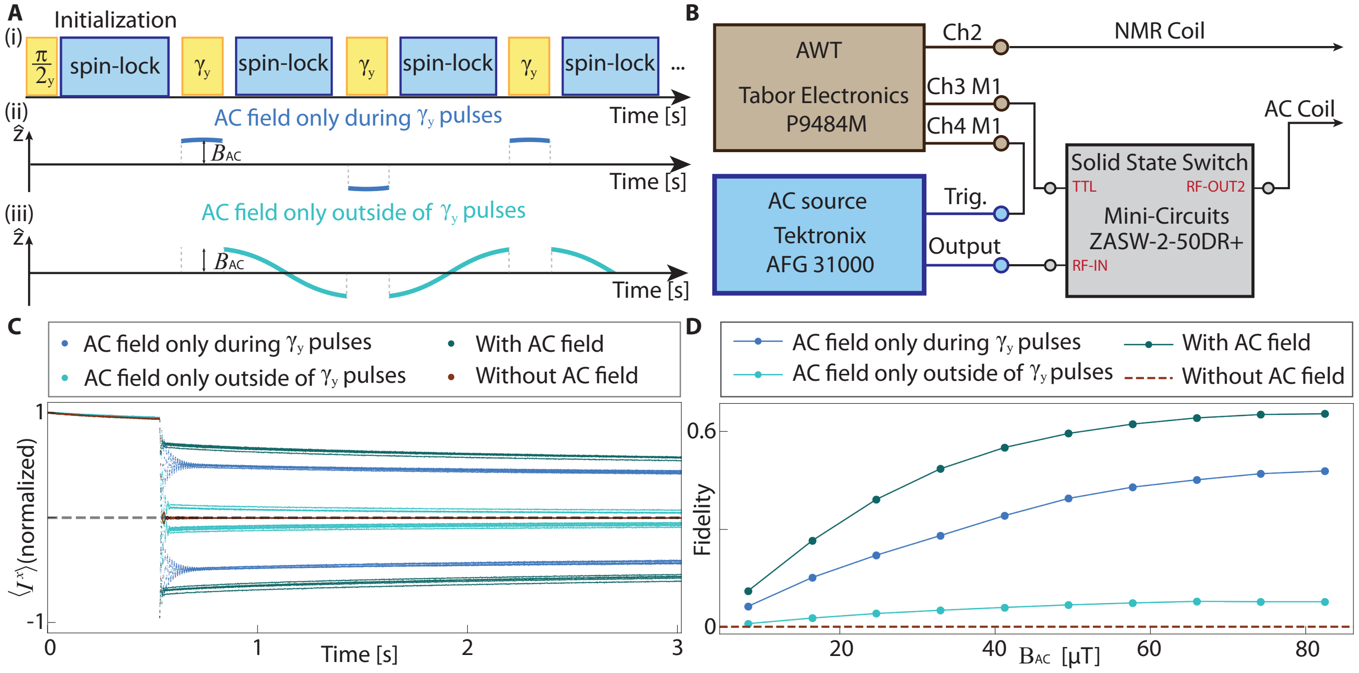

Supplementary fig 18. **AC-field only during γ_y pulses and only outside of γ_y pulses. A, Protocol:** (i) DTC sequence is applied after initializing ^{13}C nuclei in the $\hat{\mathbf{x}}$-axis by applying $(\pi/2)_y$ pulse and the spin-lock sequence (train of θ_x pulses). To examine the affect of the AC field during γ_y pulses, AC-field is applied (ii) only during γ_y pulses and (iii) only outside of γ_y pulses. **B, Apparatus:** AWT (Arbitrary Waveform Transceiver) channel 2 generates pulses sent to the NMR coil after amplification. Marker 1 of channel 4 triggers the AC source, while marker 1 of channel 3 controls a solid-state switch, allowing the AC field to reach the AC coil only when the marker signal is high. **C, Signal:** $\langle I_x \rangle$ of the ^{13}C nuclei in the diamond, normalized to the amplitude of the initial signal. Dark blue: AC field applied only during γ_y pulses; light blue: AC field applied only outside γ_y pulses; dark green: AC field applied throughout the entire DTC sequence; brown: No AC field applied. $B_{\mathrm{AC}} = 82.4\ \mu$T, θ_x pulses ($\approx 52.8\ \mu$s), γ_y pulses ($\approx 105.7\ \mu$s), and pulse separation τ ($\approx 36.0\ \mu$s). **D, Fidelity vs B_{AC}** Same color scheme as C. The fidelity of the DTC is measured while varying B_{AC}.

S9. EFFECT OF AC FIELD DURING γ_y PULSES

In Sec. S6 B, we theoretically show that the overlap between finite-length γ_y pulses and the applied AC field is responsible for the observed extended coherence of ^{13}C nuclear spins in their $\hat{\mathbf{x}}$-polarization. To confirm that this overlap is indeed the source of the extended coherence, we conduct experiments in which the AC field is applied exclusively during γ_y pulses or exclusively outside of γ_y pulses.

Supplementary fig 18A illustrates the schematic, while Supplementary fig 18B details the experimental setup. A solid-state switch (Mini-Circuits ZASW-2-50DR+) is used to gate the continuous AC field applied from the source (Tektronix AFG 31000), allowing it to be applied only during or outside γ_y pulses. We compare the fidelity of these two cases with the fidelity obtained when the AC field is applied throughout the entire DTC sequence and when no AC field is applied. Supplementary fig 18C presents the raw data (time vs. $\langle I_x \rangle$) of all four cases when $B_{\mathrm{AC}} \approx 82.4\ \mu$T, while Supplementary fig 18D shows how the fidelity changes across all four cases as B_{AC} increases. Supplementary fig 18C and D reveal that most of the increase in the fidelity originates from the overlap of the AC field with γ_y pulses, as predicted by the theory, for all values of B_{AC}. Although there is a slight increase in fidelity when applying the AC field only outside of γ_y pulses, it is minimal compared to the substantial increase observed when the AC field is applied only during γ_y pulses.

Theoretically, an increase in fidelity is not expected when the AC magnetic field is applied solely outside of the γ_y pulses. We attribute this slight increase to the θ_x pulses possessing a minor y-component in their pulse transients, with its effect accumulating over the train of θ_x pulses [51].

[1] X. Mi, M. Ippoliti, C. Quintana, A. Greene, Z. Chen, J. Gross, F. Arute, K. Arya, J. Atalaya, R. Babbush, *et al.*, Time-crystalline eigenstate order on a quantum processor, Nature **601**, 531 (2022).
[2] J. Zhang, P. W. Hess, A. Kyprianidis, P. Becker, A. Lee, J. Smith, G. Pagano, I.-D. Potirniche, A. C. Potter, A. Vishwanath, *et al.*, Observation of a discrete time crystal, Nature **543**, 217 (2017).
[3] A. Kyprianidis, F. Machado, W. Morong, P. Becker, K. S. Collins, D. V. Else, L. Feng, P. W. Hess, C. Nayak, G. Pagano, *et al.*, Observation of a prethermal discrete time crystal, Science **372**, 1192 (2021).
[4] S. Choi, J. Choi, R. Landig, G. Kucsko, H. Zhou, J. Isoya, F. Jelezko, S. Onoda, H. Sumiya, V. Khemani, *et al.*, Observation of discrete time-crystalline order in a disordered dipolar many-body system, Nature **543**, 221 (2017).
[5] J. Rovny, R. L. Blum, and S. E. Barrett, Observation of discrete-time-crystal signatures in an ordered dipolar many-body system, Phys. Rev. Lett. **120**, 180603 (2018).
[6] S. Pal, N. Nishad, T. S. Mahesh, and G. J. Sreejith, Temporal order in periodically driven spins in star-shaped clusters, Phys. Rev. Lett. **120**, 180602 (2018).
[7] A. Stasiuk and P. Cappellaro, Observation of a prethermal $u(1)$ discrete time crystal, Phys. Rev. X **13**, 041016 (2023).
[8] J. Randall, C. Bradley, F. Van Der Gronden, A. Galicia, M. Abobeih, M. Markham, D. Twitchen, F. Machado, N. Yao, and T. Taminiau, Many-body–localized discrete time crystal with a programmable spin-based quantum simulator, Science **374**, 1474 (2021).
[9] W. Beatrez, C. Fleckenstein, A. Pillai, E. de Leon Sanchez, A. Akkiraju, J. Diaz Alcala, S. Conti, P. Reshetikhin, E. Druga, M. Bukov, and A. Ajoy, Critical prethermal discrete time crystal created by two-frequency driving, Nature Physics **19**, 407 (2023).
[10] R. Zhang, W. Xiao, Y. Ding, Y. Feng, X. Peng, L. Shen, C. Sun, T. Wu, Y. Wu, Y. Yang, Z. Zheng, X. Zhang, J. Chen, and H. Guo, Recording brain activities in unshielded earth's field with optically pumped atomic magnetometers, Science Advances **6**, eaba8792 (2020), https://www.science.org/doi/pdf/10.1126/sciadv.aba8792.
[11] S. Groeger, G. Bison, J.-L. Schenker, R. Wynands, and A. Weis, A high-sensitivity laser-pumped mx magnetometer, The European Physical Journal D **38**, 239 (2006).
[12] R. Ijsselsteijn, M. Kielpinski, S. Woetzel, T. Scholtes, E. Kessler, R. Stolz, V. Schultze, and H.-G. Meyer, A full optically operated magnetometer array: An experimental study, The Review of scientific instruments **83**, 113106 (2012).
[13] K. G. Kurian, S. S. Sahoo, P. Madhu, and G. Rajalakshmi, Single-beam room-temperature atomic magnetometer with large bandwidth and dynamic range, Phys. Rev. Appl. **19**, 054040 (2023).
[14] V. G. Lucivero, P. Anielski, W. Gawlik, and M. W. Mitchell, Shot-noise-limited magnetometer with sub-picotesla sensitivity at room temperature, Review of Scientific Instruments **85**, 113108 (2014), https://pubs.aip.org/aip/rsi/article-pdf/doi/10.1063/1.4901588/13414687/113108_1_online.pdf.
[15] V. Schultze, B. Schillig, R. IJsselsteijn, T. Scholtes, S. Woetzel, and R. Stolz, An optically pumped magnetometer working in the light-shift dispersed mz mode, Sensors **17**, 10.3390/s17030561 (2017).
[16] O. Alem, K. L. Sauer, and M. V. Romalis, Spin damping in an rf atomic magnetometer, Phys. Rev. A **87**, 013413 (2013).
[17] P. D. D. Schwindt, B. Lindseth, S. Knappe, V. Shah, J. Kitching, and L.-A. Liew, Chip-scale atomic magnetometer with improved sensitivity by use of the mx technique, Applied Physics Letters **90**, 081102 (2007), https://pubs.aip.org/aip/apl/article-pdf/doi/10.1063/1.2709532/14368535/081102_1_online.pdf.
[18] S. Li, J. Liu, M. Jin, K. Tetteh Akiti, P. Dai, Z. Xu, and T. Eric-Theophilus Nwodom, A kilohertz bandwidth and sensitive scalar atomic magnetometer using an optical multipass cell, Measurement **190**, 110704 (2022).
[19] G. Chatzidrosos, A. Wickenbrock, L. Bougas, N. Leefer, T. Wu, K. Jensen, Y. Dumeige, and D. Budker, Miniature cavity-enhanced diamond magnetometer, Phys. Rev. Appl. **8**, 044019 (2017).
[20] I. Fescenko, A. Jarmola, I. Savukov, P. Kehayias, J. Smits, J. Damron, N. Ristoff, N. Mosavian, and V. M. Acosta, Diamond magnetometer enhanced by ferrite flux concentrators, Phys. Rev. Res. **2**, 023394 (2020).
[21] A. Kuwahata, T. Kitaizumi, K. Saichi, T. Sato, R. Igarashi, T. Ohshima, Y. Masuyama, T. Iwasaki, M. Hatano, F. Jelezko, M. Kusakabe, T. Yatsui, and M. Sekino, Magnetometer with nitrogen-vacancy center in a bulk diamond for detecting magnetic nanoparticles in biomedical applications, Scientific Reports **10** (2020).
[22] J. L. Webb, J. D. Clement, L. Troise, S. Ahmadi, G. J. Johansen, A. Huck, and U. L. Andersen, Nanotesla sensitivity magnetic field sensing using a compact diamond nitrogen-vacancy magnetometer, Applied Physics Letters **114**, 231103 (2019), https://pubs.aip.org/aip/apl/article-pdf/doi/10.1063/1.5095241/19767868/231103_1_online.pdf.
[23] H. Clevenson, L. M. Pham, C. Teale, K. Johnson, D. Englund, and D. Braje, Robust high-dynamic-range vector magnetometry with nitrogen-vacancy centers in diamond, Applied Physics Letters **112**, 252406 (2018), https://pubs.aip.org/aip/apl/article-pdf/doi/10.1063/1.5034216/14514188/252406_1_online.pdf.
[24] K. Jensen, N. Leefer, A. Jarmola, Y. Dumeige, V. M. Acosta, P. Kehayias, B. Patton, and D. Budker, Cavity-enhanced room-temperature magnetometry using absorption by nitrogen-vacancy centers in diamond, Phys. Rev. Lett. **112**, 160802 (2014).
[25] J. F. Barry, M. J. Turner, J. M. Schloss, D. R. Glenn, Y. Song, M. D. Lukin, H. Park, and R. L. Walsworth, Optical magnetic detection of single-neuron action potentials using quantum defects in diamond, Proceedings of the National Academy of Sciences **113**, 14133 (2016), https://www.pnas.org/doi/pdf/10.1073/pnas.1601513113.
[26] T. Wolf, P. Neumann, K. Nakamura, H. Sumiya, T. Ohshima, J. Isoya, and J. Wrachtrup, Subpicotesla diamond magnetometry, Phys. Rev. X **5**, 041001 (2015).
[27] P. Maletinsky, S. Hong, M. Grinolds, B. Hausmann, M. Lukin, M. Loncar, and A. Yacoby, A robust scanning diamond sensor for nanoscale imaging with single nitrogen-vacancy centres, Nature nanotechnology **7**, 320 (2012).
[28] K. Yahata, Y. Matsuzaki, S. Saito, H. Watanabe, and J. Ishi-Hayase, Demonstration of vector magnetic field sensing by simultaneous control of nitrogen-vacancy centers in diamond using multi-frequency microwave pulses, Applied Physics Letters **114**, 022404 (2019), https://pubs.aip.org/aip/apl/article-pdf/doi/10.1063/1.5079925/14520954/022404_1_online.pdf.
[29] *High Temperature Fluxgate Probes*, Bartington Instruments ().
[30] *DRV425 Fluxgate Magnetic-Field Sensor*, Texas Instruments (2015).

[31] *Compact 3-Axis Magnetometers THM1176 and TFM1186*, Metrolab Technology ().
[32] N. Haned and M. Missous, Nano-tesla magnetic field magnetometry using an ingaas–algaas–gaas 2deg hall sensor, Sensors and Actuators A: Physical **102**, 216 (2003).
[33] M. Díaz-Michelena, Small magnetic sensors for space applications, Sensors **9**, 2271 (2009).
[34] C. Coillot, J. Moutoussamy, R. Lebourgeois, S. Ruocco, and G. Chanteur, Principle and Performance of a Dual-Band Search Coil Magnetometer: A New Instrument to Investigate Fluctuating Magnetic Fields in Space, IEEE Sensors Journal **10**, 255 (2009).
[35] J. F. Barry, R. A. Irion, M. H. Steinecker, D. K. Freeman, J. J. Kedziora, R. G. Wilcox, and D. A. Braje, Ferrimagnetic oscillator magnetometer, Phys. Rev. Appl. **19**, 044044 (2023).
[36] O. Sahin, H. A. Asadi, P. Schindler, A. Pillai, E. Sanchez, M. Markham, M. Elo, M. McAllister, E. Druga, C. Fleckenstein, M. Bukov, and A. Ajoy, Continuously tracked, stable, large excursion trajectories of dipolar coupled nuclear spins (2022), arXiv:2206.14945 [quant-ph].
[37] W. Beatrez, A. Pillai, O. Janes, D. Suter, and A. Ajoy, Electron induced nanoscale nuclear spin relaxation probed by hyperpolarization injection, Phys. Rev. Lett. **131**, 010802 (2023).
[38] O. Sahin, E. de Leon Sanchez, S. Conti, A. Akkiraju, P. Reshetikhin, E. Druga, A. Aggarwal, B. Gilbert, S. Bhave, and A. Ajoy, High field magnetometry with hyperpolarized nuclear spins, Nature communications **13**, 5486 (2022).
[39] P. Weinberg and M. Bukov, QuSpin: a Python package for dynamics and exact diagonalisation of quantum many body systems part I: spin chains, SciPost Phys. **2**, 003 (2017).
[40] P. Weinberg and M. Bukov, QuSpin: a Python package for dynamics and exact diagonalisation of quantum many body systems. Part II: bosons, fermions and higher spins, SciPost Phys. **7**, 020 (2019).
[41] K. Harkins, C. Fleckenstein, N. D'Souza, P. M. Schindler, D. Marchiori, C. Artiaco, Q. Reynard-Feytis, U. Basumallick, W. Beatrez, A. Pillai, M. Hagn, A. Nayak, S. Breuer, X. Lv, M. McAllister, P. Reshetikhin, E. Druga, M. Bukov, and A. Ajoy, Nanoscale engineering and dynamical stabilization of mesoscopic spin textures (2023), arXiv:2310.05635 [quant-ph].
[42] L. J. I. Moon, P. M. Schindler, Y. Sun, E. Druga, J. Knolle, R. Moessner, H. Zhao, M. Bukov, and A. Ajoy, Experimental observation of a time rondeau crystal: Temporal disorder in spatiotemporal order (2024), arXiv:2404.05620 [quant-ph].
[43] D. J. Luitz, R. Moessner, S. L. Sondhi, and V. Khemani, Prethermalization without temperature, Phys. Rev. X **10**, 021046 (2020).
[44] C. Fleckenstein and M. Bukov, Thermalization and prethermalization in periodically kicked quantum spin chains, Phys. Rev. B **103**, 144307 (2021).
[45] C. Fleckenstein and M. Bukov, Prethermalization and thermalization in periodically driven many-body systems away from the high-frequency limit, Phys. Rev. B **103**, L140302 (2021).
[46] D. A. Abanin, W. De Roeck, and F. m. c. Huveneers, Exponentially slow heating in periodically driven many-body systems, Phys. Rev. Lett. **115**, 256803 (2015).
[47] T. Mori, T. Kuwahara, and K. Saito, Rigorous bound on energy absorption and generic relaxation in periodically driven quantum systems, Phys. Rev. Lett. **116**, 120401 (2016).
[48] A. Pizzi, A. Nunnenkamp, and J. Knolle, Classical prethermal phases of matter, Phys. Rev. Lett. **127**, 140602 (2021).
[49] W. W. Ho, I. Protopopov, and D. A. Abanin, Bounds on energy absorption and prethermalization in quantum systems with long-range interactions, Phys. Rev. Lett. **120**, 200601 (2018).
[50] W. Beatrez, O. Janes, A. Akkiraju, A. Pillai, A. Oddo, P. Reshetikhin, E. Druga, M. McAllister, M. Elo, B. Gilbert, D. Suter, and A. Ajoy, Floquet prethermalization with lifetime exceeding 90 s in a bulk hyperpolarized solid, Phys. Rev. Lett. **127**, 170603 (2021).
[51] M. Mehring and J. S. Waugh, Phase transients in pulsed nmr spectrometers, Review of Scientific Instruments **43**, 649 (1972).
